# Food insecurity and its related indicators in an Iranian population: An application of machine learning methods in data from Golestan Province

**DOI:** 10.1371/journal.pone.0361131

**Published:** 2026-10-30

**Authors:** Fatemeh Nassaj-Torshizi, Farid Zayeri, Nasser Behnampour, Mohammad-Reza Honarvar, Morteza Mohammadzadeh

**Affiliations:** 1 Department of Biostatistics, School of Allied Medical Sciences, Shahid Beheshti University of Medical Sciences, Tehran, Iran; 2 Proteomics Research Center, Department of Biostatistics, School of Allied Medical Sciences, Shahid Beheshti University of Medical Sciences, Tehran, Iran; 3 Health Management and Social Development Research Centre, Department of Biostatistics and Epidemiology, Golestan University of Medical Sciences, Gorgan, Islamic Republic of Iran; 4 Health Management and Social Development Research Center, Department of Nutrition, Golestan University of Medical Sciences, Gorgan, Islamic Republic of Iran; 5 Department of Biostatistics, School of Public Health, Iran University of Medical Sciences, Tehran, Iran; International Islamic University Malaysia, MALAYSIA

## Abstract

**Background:**

Household food insecurity (FI) is a major public health concern associated with socioeconomic vulnerability. Unfortunately, the prevalence of food insecurity in Iran has increased substantially in recent years due to the economic challenges associated with prolonged international sanctions. In this context, this study aimed to estimate the prevalence of household FI in northern Iran and compare the predictive performance of traditional and machine learning algorithms for classifying households according to presence of FI.

**Methods:**

This cross-sectional study included 4,948 households from Golestan Province in north of Iran. The study sample was selected using the multistage cluster sampling between December 2021 and September 2022. FI was assessed using the Household Food Insecurity Access Scale. The data was analyzed using the logistic regression, random forest, support vector machine, decision tree, naïve Bayes, and artificial neural network models. Model performance was assessed using the area under the receiver operating characteristic curve (AUC), sensitivity, specificity, accuracy, balanced accuracy, Brier score, calibration plots, and DeLong’s test for pairwise AUC comparisons. The bootstrap resampling was applied to assess the stability and uncertainty of predictor importance. In addition, the estimated rankings derived from the logistic regression and random forest models were compared using the Spearman’s correlation coefficient.

**Results:**

The prevalence of household FI was 61.7%, including 32.3% mild, 20.3% moderate, and 9.1% severe FI. The multivariable logistic regression analysis showed that lower income, lower educational attainment, unemployment, lack of insurance coverage, absence of supplementary insurance, lack of car or house ownership, fewer employed household members, larger household size, being under coverage of supportive organizations, having incurable disease, and Turkmen ethnicity remained associated with FI. The pairwise DeLong’s test showed modest differences between the fitted models, with no statistically significant difference between the logistic regression and either neural network or SVM radial models after Bonferroni correction. Household income was ranked as the most important predictor in both the logistic regression and random forest models.

**Conclusions:**

In general, our estimates revealed that household FI was highly prevalent in the sample under study and strongly associated with socioeconomic disadvantage. Logistic regression, neural network, and SVM models demonstrated relatively similar predictive performance, while more complex ML approaches did not show substantial practical improvement over conventional methods in this dataset. Given the serious consequences of household food insecurity, health policymakers should pay more attention to improve socioeconomic conditions of households residing in the studied areas.

## Introduction

Food insecurity (FI), widely defined as a household-level economic or social condition of limited or uncertain access to adequate food, is a major issue for public health and global development. This problem not only has a detrimental effect on people’s well-being and productivity but also is one of the major public health problems in all countries regardless of their socioeconomic status. These circumstances may lead to a vicious cycle in which households are unable to access or produce enough food [1–3].

According to the reports in 2023, approximately two billion people (about 28.9% of the world’s population) experience moderate to severe FI [4]. This means that they have limited access to enough nourishing food. The prevalence of moderate or severe FI in Africa was reported as 58.0%, which is nearly double the global average. However, lower prevalence rates were reported in Latin America and the Caribbean, Asia, and Oceania compared with Africa (8.2, 24.8 and 26.8 percent, respectively) [4]. FI is also a major public health concern in Iran. Some reports in 2016 show that 49% of Iranian households experienced levels of FI. Regarding these reports, international sanctions have seemingly increased the prevalence of FI in recent years [5,6]. Another study in Golestan Province, northern Iran, also showed that 56.8% households in this region experienced some levels of FI in 2016 [7].

FI and its consequences are affected by a combination of factors, such as severe climate change, regional conflicts and economic crises which result in unemployment, inflation, and consequently a shortage of nutrient-dense food [8,9]. Previous studies have demonstrated that lower levels of socioeconomic status characterized by unemployment, low income, and limited educational attainment as well as some demographic factors, such as single adults, single-parent households with children, and female-headed households, are significantly associated with a higher risk of FI [10–14]. Regarding these results, FI is fundamentally linked to economic resources and is disproportionately prevalent among marginalized and vulnerable populations [10].

To identify factors associated with FI, researchers have previously applied a variety of statistical methods in different datasets. These methods can be classified into two main categories: traditional and machine learning-based approaches. In this context, well-known discrimination and classification methods as well as logistic regression models are the most common approaches of the conventional statistical approaches. With advances in software and computational methods, machine learning algorithms, such as decision tree analysis, artificial neural networks, and support vector machines (SVM), have increasingly been applied in public health research. These methods may help capture complex interactions and nonlinear relationships in some datasets [15]. Nevertheless, the predictive performance of each method can vary depending on the specific characteristics of the dataset under study.

Several studies have applied ML algorithms to investigate factors associated with FI. For instance, a study by Qasrawi et al in 2020 evaluated seven ML methods in an Arab population and reported acceptable predictive performance for ensemble models, particularly gradient boosting and random forest, in predicting FI [15].

However, although previous studies in Iran have investigated factors associated with household food insecurity using conventional statistical approaches, evidence regarding the comparative application of conventional methods and machine learning algorithms for classifying households according to food insecurity in large population-based datasets remains limited in the published literature [7,16]. Because the predictive performance of analytical approaches may vary depending on dataset characteristics and underlying variable relationships, evaluating different modeling strategies may help us to improve understanding of household food insecurity patterns in vulnerable populations. Therefore, the present study aimed to estimate the prevalence of household food insecurity in northern Iran and to compare the predictive performance of conventional statistical methods as well as several machine learning algorithms for identifying candidate predictors of food insecurity and classifying households according to these predictors in a large population-based dataset. The findings may contribute to the existing evidence regarding the applicability of different predictive modeling strategies for household food insecurity research in the similar populations.

## Methods

### Study design

The current research is a cross-sectional study, carried out from December 2021 to September 2022, on 4948 households in Golestan province, north of Iran. This study was designed to identify the prevalence and related indicators of FI among the resident households in this province.

#### Sample size and sampling technique.

The sample size of the present study as calculated according to the main objective of the original proposal (determining the prevalence rate of FI). In this context, regarding an estimated prevalence rate of 56.8% in previous studies [7], and confidence level of 95%, a sample size of at least 4864 was needed according to the Cochrane formula ($n=\frac{N{\left[Z_{\text{1}-\frac{\alpha }{\text{2}}}\right]}^{\text{2}}\left[P(\text{1}-P)\right]}{(N-\text{1})[\varepsilon {]}^{\text{2}}+{\left[Z_{\text{1}-\frac{\alpha }{\text{2}}}\right]}^{\text{2}}\left[P(\text{1}-P)\right]}$).

A multistage cluster sampling design was employed to recruit the study population from Golestan Province, Iran. At the first stage, all 14 counties (each county includes 1 urban and related rural areas) of the Golestan province were considered as the primary clusters. In the next stage, the sample size was allocated to each county according to the proportion of the population of the county (PPS approach). Again, using the PPS approach, the sample size of each county were allocated to the rural and urban areas according to the proportion of the rural and urban populations. In each of 14 urban areas (cities), the list of comprehensive urban health centers was provided and then a number of these health center were randomly chosen using the systematic sampling approach. In each of the selected center, the list of undercover households was provided and a sample of households was randomly extracted. To sample from rural areas, first the list of rural areas related to each 14 counties was provided and then a number of rural areas were randomly chosen within each county. In each of the selected rural areas, the list of undercover household of the comprehensive rural health center was provided and then a number of households were randomly chosen within each center. In all of sampling stages, the PPS methodology was considered for allocating the total sample size to the urban and rural households.

### Main outcome and predictors under study

In the present study, the Household FI Access Scale (HFIAS) was used to assess the households’ FI status. The Persian version of the HFIAS has previously undergone cultural adaptation and psychometric validation in Iranian population, demonstrating acceptable validity and reliability in prior studies [17]. The HFIAS index was first developed by the Food and Nutrition Technical Assistance II (FANTA) project between 2001 and 2006 (18). This index is based on a short-form questionnaire including nine items (each item with a Likert scale of 0 = never, 1 = rarely, 2 = sometimes, and 3 = often) for assessing different indicators of FI within the previous month. For each household under study, the total score of HFIAS can be classified as 0–1, 2–7, 8–14, and 15–27 indicating food security, mild FI, moderate FI, and severe FI, respectively [18]. This four-level classification shows the severity of FI for each household. In addition, we used a binary outcome of FI (0 = food security, 1 = any level of FI) in the data analysis process. During the data gathering process, our priority was asking the questions of the HFIAS from the mothers in each selected household. If this was not possible, the questions were asked from the person with adequate information about the consumed food of household during the previous month. The items of the HFIAS were completed by the trained interviewers for each household and after double-checking of items a random subsample, the data was entered in the Golestan SAMAT database [19].

In statistical analysis, a number of demographic, socioeconomic, household-related, and health-related characteristics were considered as the candidate predictors of household food insecurity. Some of these predictors were related to the head of household including: age, gender, residence place (urban or rural), marital status, occupational status, ethnicity, primary and supplementary insurance status, and education level. Other predictors were associated with the household, i.e., number of household members, number of employed members, cash subsidy status, being under cover of supporting organizations, having incurable disease, having COVID-19, car ownership, house ownership, and income (S1 Table).

### Statistical analysis

The binary outcome variable, food insecurity status, was recoded as “Secure” and “Insecure.” Predictor variables included demographic, socioeconomic, and health-related characteristics. Missing data were handled using complete-case analysis (missing rate was below 6% for all variables) after inspection of missingness patterns [20,21]. Prior to fitting statistical models, patterns of missingness were explored descriptively. The proportion of missing data was low (<6% for all variables), and no systematic differences in missingness were observed across the main outcome categories or key demographic variables. Given the limited extent of missing data and the absence of evidence suggesting substantial informative missingness, complete-case analysis was considered an acceptable approach for the present study.

To describe the binary and ordinal outcome under study, the prevalence of FI and levels of severity of FI was reported. At first step of data analysis, we evaluated the relationship between the binary outcomes (presence of FI in each household) with categorical predictors using the chi-square test.

Regarding the sampling technique, the data were weighted to adjust for sample selection and non-response [22]. In this context, the weighting factor (W) consisted of the following formula:


$$\begin{array}{c} \text{W}={\text{W}}_{\text{1}}\times {\text{W}}_{\text{2}}\times {\text{f}}_{\text{1}}, \end{array}$$

where ${\text{W}}_{\text{1}}$ is the inverse of the probability of selection for each rural area related to each county, ${\text{W}}_{\text{2}}$ is the inverse of the probability of selection for each household within each comprehensive urban or rural health center, ${\text{f}}_{\text{1}}$ is non-response adjustment factor calculated by comprehensive urban or rural health center (household level).

Using the described weighting factor, univariable logistic regression analyses were performed. Subsequently, multivariable logistic regression analysis was conducted using forward stepwise variable selection based on the Akaike Information Criterion (AIC). Adjusted odds ratios with corresponding 95% confidence intervals were extracted from the final selected model.

The analytical workflow included data preprocessing, stratified train–test splitting, repeated cross-validation, hyperparameter tuning, independent test-set evaluation, calibration assessment, and model comparison. Because the primary objective of the study was predictive modeling rather than causal inference, machine learning algorithms with different underlying structures were evaluated and compared.

In this study, the following models were implemented: logistic regression, random forest, support vector machine with radial basis kernel, decision tree, naïve Bayes, and artificial neural network [23–25]. No artificial class balancing techniques, such as over-sampling, under-sampling, or synthetic minority over-sampling technique (SMOTE), were applied. Instead, the original class distribution was preserved to maintain the real-world distribution of food insecurity outcomes [26,27].

The dataset was randomly divided into training (70%) and testing (30%) subsets using stratified sampling to preserve the class distribution of the outcome variable in both datasets [28]. The hold-out test set was used for internal validation because both the training and testing datasets were derived from the same source population and sampling framework. To reduce the risk of overfitting, all hyperparameter tuning procedures were conducted exclusively within the repeated cross-validation framework of the training dataset, while the final model evaluation was performed on a fully independent test dataset. Model development and hyperparameter tuning were conducted exclusively within the training dataset using repeated stratified 5-fold cross-validation with three repetitions, following established predictive modeling recommendations [28]. Area under the receiver operating characteristic curve (AUC) was also used as the primary optimization metric during model training because of its robustness in evaluating binary classification performance [29,30].

Hyperparameter tuning was conducted using predefined tuning grids for each of the applied models. For random forest model, the number of variables, which randomly sampled at each split (mtry), was tuned while the number of trees was fixed at 500. For support vector machine model, sigma and cost parameters were optimized. Also, the decision tree algorithm was tuned using the complexity parameter. Laplace smoothing was optimized without applying kernel density estimation in naïve Bayes model, and optimization focused on the number of hidden units and weight decay parameters were used for the neural network model. Predictor centering and scaling were applied only for support vector machine and neural network models within the resampling procedure [28].

Final predictive performance was evaluated only on the independent internal test dataset. External validation using an independent population was not performed in the present study. Discriminative performance was assessed using AUC with 95% confidence intervals. Additional performance measures included sensitivity, specificity, positive predictive value (PPV), negative predictive value (NPV), accuracy, balanced accuracy, and Brier score. The receiver operating characteristic (ROC) curves were generated for all models. Calibration was assessed using the decile-based calibration plots comparing the mean predicted probabilities with the observed proportions of food insecurity across deciles of predicted risk. In addition to calibration plots and Brier scores, model calibration was quantitatively assessed using calibration intercepts and slopes calculated on the independent test dataset. Calibration intercepts evaluate systematic over- or underestimation of predicted probabilities, with an ideal value of 0, whereas calibration slopes assess the agreement between predicted and observed risks, with an ideal value of 1. Together, these measures provide a comprehensive assessment of calibration beyond graphical evaluation alone. A fixed classification threshold of 0.61 was used for all classification models to reflect the approximate observed prevalence of the food insecurity in the study population [29–31]. A common classification threshold of 0.61, corresponding approximately to the observed prevalence of food insecurity in the study population, was applied across all models when calculating threshold-dependent performance measures, including sensitivity, specificity, positive predictive value, negative predictive value, and accuracy. The purpose of using a common threshold was to facilitate direct comparison of classification performance across competing models. Model discrimination was primarily evaluated using the Youden index in ROC curve analysis. Pairwise comparisons of AUC values between models were also performed using the DeLong’s test. In this test, P-values were adjusted using the Bonferroni method [32].

To assess predictors associated with food insecurity within the conventional statistical framework, forward stepwise multivariable logistic regression was performed using the training dataset. Adjusted odds ratios and corresponding 95% confidence intervals were reported for the final model according to the described weighting factors [33]. The results of conventional and machine learning approaches were compared regarding the obtained variable importance from the multivariable logistic regression and random forest models. For logistic regression, predictor importance was quantified using the absolute value of the Wald z statistic derived from the final forward stepwise logistic model. To assess the stability and uncertainty of logistic regression variable importance, bootstrap resampling with 200 iterations was performed. In each bootstrap sample, forward stepwise logistic regression based on the Akaike Information Criterion (AIC) was repeated.

For the random forest model, variable importance was assessed using the mean decrease in Gini impurity obtained from the final tuned model. To quantify uncertainty in random forest importance estimates, nonparametric bootstrap resampling with 200 iterations was conducted. In each bootstrap sample, a random forest model was refitted using the optimal hyperparameters identified during model tuning, and variable importance measures were recalculated. To evaluate agreement between predictor rankings obtained from the logistic regression and random forest models, the Spearman’s correlation coefficient was calculated using predictor-level importance ranks.

All statistical and machine learning analyses were performed in R software using a reproducible analytical workflow. The main R packages used included haven, dplyr, forcats, caret, pROC, randomForest, rpart, klaR, nnet, ggplot2, broom, purrr, readr, tibble, e1071, and MASS. The dataset was initially imported from SPSS format, and labelled variables were converted to categorical factors while preserving their original labels. In addition, the STATA software, version 14.0, was used to account for weighting factor in the statistical analysis.

### Ethical approval

This study was performed using the data obtained from a previously approved research project conducted at Golestan University of Medical Sciences (Approval No:IR.GOUMS.REC.1394.265).

The use of these data for the present analysis was also approved by the Ethics Committee of Shahid Beheshti University of Medical Sciences (Approval No:IR.SBMU.RETECH.REC.1404.828).

## Results

Based on the findings, among 4,948 surveyed households, the prevalence of household FI was 61.7% (3,053 households), including 32.3% (1,597 households) with mild, 20.3% (1,004 households) with moderate, and 9.1% (452 households) with severe FI.

The chi-square analyses demonstrated significant differences in food insecurity across most household characteristics. Food insecurity was more prevalent among households residing in rural areas (67.2%), households with female heads (70.9%), households with unmarried heads (70.9%), households with unemployed heads (82.1%) and households without employed members (75.8%) and households without basic or supplementary insurance (respectively, 79.6% and 67.5%). Food insecurity was more common among households with lower educational levels (75.9%), lower levels of income (85.6%), those with a member having an incurable disease (75.1%), households without car ownership (75.0%) or house ownership (70.1%), households receiving cash subsidies (68.2%), and those who are dependent on supportive organizations (84.6%). The highest proportion of food-secure households was observed among households with Fars ethnicity (42.9%). Although food security was relatively more frequent among households with older heads (39.1%) and households with fewer than four members (38.8%), these differences were not statistically significant. Significant associations with food insecurity were observed for most evaluated household characteristics (P < 0.001), whereas no statistically significant associations were observed for age of household head (P = 0.323), number of household members (P = 0.496), or a history of COVID-19 infection (P = 0.074). Table 1 displays more details about these findings.

**Table 1:** Univariate relationship between characteristics of households and FI.

Characteristic of household	Category	Food Insecurity		P
		Secure	Insecure	
**Residence place**	Urban	1130 (43.2)	1484 (56.8)	<0.001
	Rural	765 (32.8)	1569 (67.2)	
**Age of the head**	Less than 50	1109 (37.7)	1830 (62.3)	0.323
	50 or more	786 (39.1)	1223 (60.9)	
**Gender of the head**	Male	1732 (39.5)	2655 (60.5)	<0.001
	Female	163 (29.1)	398 (70.9)	
**Marital Status of the head**	Married	1707 (39.5)	2611 (60.5)	<0.001
	Other	179 (29.1)	437 (70.9)	
**Ethnicity of the head**	Fars	871 (42.9)	1161 (57.1)	<0.001
	Turkmen	732 (39.2)	1133 (60.8)	
	Other	292 (27.8)	759 (72.2)	
**Number of household members**	Less than 4	815 (38.8)	1283 (61.2)	0.496
	4 or more	1080 (37.9)	1770 (62.1)	
**Number of employed**	2 or more person	374 (59.1)	259 (40.9)	<0.001
	1 person	1304 (38.2)	2113 (61.8)	
	None	217 (24.2)	681 (75.8)	
**Education level**	Illiterate	184 (24.1)	580 (75.9)	<0.001
	Primary or middle school	509 (29.1)	1241 (70.9)	
	High school	164 (31.2)	362 (68.8)	
	Diploma	461 (45.2)	560 (54.8)	
	University degree	576 (65.2)	308 (34.8)	
**Occupational status**	Unemployed	139 (17.9)	637 (82.1)	<0.001
	Laborer	221 (21.4)	810 (78.6)	
	Farmer/Rancher	396 (41.9)	549 (58.1)	
	Self-employed	454 (46.3)	527 (53.7)	
	Jobless/Retired	300 (50.9)	289 (49.1)	
	Public sector employees	373 (63.3)	216 (36.7)	
**Insurance**	Yes	1760 (40.9)	2542 (59.1)	<0.001
	No	129 (20.4)	502 (79.6)	
**Supplementary insurance**	Yes	585 (61.4)	367 (38.6)	<0.001
	No	1283 (32.5)	2667 (67.5)	
**Cash subsidy**	Received	1111 (31.8)	2381 (68.2)	<0.001
	Not received	784 (53.8)	672 (46.2)	
**Under cover of supportive organizations**	Yes	105 (15.4)	579 (84.6)	<0.001
	No	1790 (42.0)	2474 (58.0)	
**Having incurable disease**	Yes	144 (24.9)	435 (75.1)	<0.001
	No	1751 (40.1)	2618 (59.9)	
**Having COVID-19**	Yes	663 (40.0)	993 (60.0)	0.074
	No	1232 (37.4)	2060 (62.6)	
**Car ownership**	Yes	1238 (52.0)	1141 (48.0)	<0.001
	No	632 (25.0)	1891 (75.0)	
**House ownership**	Personal	1609 (41.3)	2284 (58.7)	<0.001
	Rental	208 (29.9)	487 (70.1)	
	Other	71 (21.0)	267 (79.0)	
**Income**	Very low	126 (14.4)	746 (85.6)	<0.001
	Low	267 (27.9)	690 (72.1)	
	Medium	286 (31.4)	624 (68.6)	
	High	538 (46.4)	621 (53.6)	
	Very high	622 (70.0)	266 (30.0)	

In the univariable logistic regression analyses, most socioeconomic and household characteristics were significantly associated with food insecurity. Statistically significant associations were observed for lower household income, unemployment, lower educational attainment, lack of insurance coverage, absence of supplementary insurance, lack of car ownership, and being under the coverage of supporting organizations with food insecurity. After adjustment in the forward stepwise multivariable logistic regression model, 12 predictors (including income, educational levels, levels of occupation, having 4 or more household members, households with fewer employed members, lack of car ownership, non-personal housing ownership status, absence of supplementary insurance, lack of insurance coverage, being under the coverage of supporting organizations, having an incurable disease and Turkmen households) still remained associated with food insecurity (Table 2).

**Table 2:** Results of the univariate and forward stepwise multivariable logistic regression analyses for identifying predictors of household food insecurity.

Characteristic of household	Category	Univariate		Multivariable	
		OR (95% CI)	P	OR (95% CI)	P
**Residence place**	Rural	1.528 (1.356,1.722)	< 0.001		
	Urban	Reference category			
**Age of the head**	Less than 50	1.046 (0.928, 1.180)	0.460		
	50 or more	Reference category			
**Gender of the head**	Female	1.632 (1.338, 1,999)	< 0.001		
	Male	Reference category			
**Marital Status of the head**	Other	1.613 (1.334, 1.958)	< 0.001		
	Married	Reference category			
**Ethnicity of the head**	Other	1.939 (1.642, 2.295)	< 0.001	0.966 (0.792, 1.178)	0.734
	Turkmen	1.168 (1.024, 1.332)	0.021	0.635 (0.537, 0.751)	< 0.001
	Fars	Reference category	Reference category		
**Number of household members**	4 or more	1.045 (0.928, 1.178)	0.466	1.345 (1.158, 1.562)	< 0.001
	Less than 4	Reference category	Reference category		
**Number of employed**	None	4.581 (3.655, 5.757)	< 0.001	2.014 (1.515, 2.677)	< 0.001
	1 person	2.370 (1.986, 2.833)	< 0.001	1.762 (1.437, 2.161)	< 0.001
	2 or more person	Reference category		Reference category	
**Education level**	Illiterate	6.178 (4.945, 7.750)	< 0.001	1.494 (1.106, 2.017)	0.009
	Primary or middle school	4.635 (3.878, 5.551)	< 0.001	1.796 (1.418, 2.275)	< 0.001
	High school	4.560 (3.590, 5.816)	< 0.001	2.041 (1.527, 2.728)	< 0.001
	Diploma	2.341 (1.932, 2.841)	< 0.001	1.402 (1.115, 1.763)	0.004
	University degree	Reference category	Reference category		
**Occupational status**	Unemployed	8.409 (6.495, 10.953)	< 0.001	1.522 (1.085, 2.137)	0.015
	Laborer	6.398 (5.085, 8.078)	< 0.001	1.661 (1.232, 2.238)	0.001
	Farmer/Rancher	2.339 (1.884, 2.911)	< 0.001	0.740 (0.556, 0.986)	0.040
	Self-employed	1.983 (1.599, 2.464)	< 0.001	0.842 (0.645, 1.100)	0.207
	Jobless/Retired	1.675 (1.320, 2.128)	< 0.001	1.198 (0.893, 1.606)	0.229
	Public sector employees	Reference category	Reference category		
**Insurance**	No	2.772 (2.248, 3.446)	< 0.001	1.648 (1.291, 2.102)	< 0.001
	Yes	Reference category	Reference category		
**Supplementary insurance**	No	3.334 (2.876, 3.871)	< 0.001	1.595 (1.301, 1.954)	< 0.001
	Yes	Reference category	Reference category		
**Cash subsidy**	Received	2.525 (2.221, 2.871)	< 0.001		
	Not received	Reference category			
**Under cover of supporting organizations**	Yes	4.000 (3.209, 5.036)	< 0.001	1.889 (1.454, 2.456)	< 0.001
	No	Reference category	Reference category		
**Having incurable disease**	Yes	1.992 (1.631, 2.447)	< 0.001	1.477 (1.166, 1.871)	0.001
	No	Reference category	Reference category		
**Having COVID-19**	No	1.143 (1.010, 1.294)	0.035		
	Yes	Reference category			
**Car ownership**	No	3.299 (2.915, 3.737)	< 0.001	1.542 (1.325, 1.794)	< 0.001
	Yes	Reference category	Reference category		
**House ownership**	Other	2.756 (2.067, 3.733)	< 0.001	1.728 (1.239, 2.411)	0.001
	Rental	1.638 (1.359, 1.981)	< 0.001	1.784 (1.423, 2.237)	< 0.001
	Personal	Reference category		Reference category	
**Income**	Very low	13.500 (10.660, 17.209)	< 0.001	5.047 (3.781, 6.736)	< 0.001
	Low	5.875 (4.798, 7.215)	< 0.001	3.183 (2.507, 4.042)	< 0.001
	Medium	5.060 (4.135, 6.207)	< 0.001	2.853 (2.239, 3.635)	< 0.001
	High	2.736 (2.272, 3.300)	< 0.001	1.902 (1.541, 2.347)	< 0.001
	Very high	Reference category		Reference category	

Table 3 presents the predictive performance of the evaluated models on the independent test dataset using a fixed prevalence-based classification threshold of 0.61 (prevalence rate of FI in sample under study). Overall, the neural network achieved the highest AUC, although its performance was very similar to that of logistic regression. The support vector machine (SVM) model showed slightly higher overall classification performance in terms of accuracy (0.717). This model also resulted in the highest sensitivity (0.762) among all evaluated models. However, its specificity was lower than that of the neural network and logistic regression models. The random forest model demonstrated moderate discriminative ability (AUC = 0.758) but showed lower calibration performance, reflected by a higher Brier score compared with logistic regression and neural network models. Naïve Bayes and decision tree models showed lower AUC values and larger deviations from the reference calibration line compared with other evaluated models. The neural network and logistic regression models yielded the lowest Brier scores, suggesting relatively closer agreement between predicted probabilities and observed food insecurity outcome. Positive predictive values were generally higher than negative predictive values across models, consistent with the relatively high prevalence of food insecurity in the study population. Overall, relatively similar predictive performance patterns were observed across logistic regression and the machine learning approaches. The calibration intercepts were generally close to zero for Logistic Regression, Neural Network, SVM Radial, and Random Forest, suggesting limited systematic overestimation or underestimation of predicted probabilities. Calibration slopes for Logistic Regression (0.934; 95% CI: 0.818–1.050), Neural Network (0.935; 95% CI: 0.819–1.050), and SVM Radial (0.965; 95% CI: 0.844–1.090) were relatively close to the ideal value of one. In contrast, Random Forest, Decision Tree, and Naïve Bayes showed greater departures from the ideal calibration slope (S2 Table). The estimated ROC curve from different classification model were shown in Fig 1. Pairwise comparisons of model discrimination were performed using DeLong’s test with Bonferroni correction for multiple comparisons. Several statistically significant differences in AUC values were observed between models; however, the magnitude of these differences was generally modest. Logistic regression showed different performance compared with the random forest, decision tree, and naïve Bayes models after multiple-comparison correction, whereas no statistically significant difference was observed between logistic regression and either the SVM radial or neural network models. Similarly, the neural network and SVM radial models demonstrated different performance compared with some lower-performing models, particularly decision tree and naïve Bayes. In contrast, no statistically significant differences were identified between other models, including logistic regression versus neural network and SVM radial versus neural network. Overall, these findings suggest that the utilized modeling approaches have resulted in rather similar predictive performance in this dataset. Although some significant pairwise differences were detected, these significances may be attributed to large sample of data, thus the results do not support an advantage of more complex machine learning algorithms over conventional logistic regression in this dataset (Table 4).

**Table 3:** Predictive performance indices for different models under study.

Classification methods	AUC (95% CI)	SEN	SPE	PPV	NPV	ACC	Balanced ACC	Brier Score
**Neural network**	0.777 (0.752, 0.801)	0. 718	0.693	0.792	0.602	0.708	0.706	0.184
**Logistic Regression**	0.777 (0.752, 0.801)	0. 718	0.688	0.789	0.600	0.706	0.703	0.184
**Support vector machine**	0.772 (0.747, 0.797)	0.762	0.643	0.776	0.624	0.716	0.702	0.186
**Random Forest**	0.758 (0.732, 0.783)	0.747	0.618	0.761	0.600	0.698	0.683	0.201
**Naïve Bayes**	0.746 (0.720, 0.772)	0.619	0.754	0.803	0.549	0.671	0.687	0.276
**Decision Tree**	0.734 (0.708, 0.761)	0.751	0.575	0.742	0.587	0.684	0.663	0.208

Abbreviations: AUC, area under the receiver operating characteristic curve; CI, confidence interval; SEN, sensitivity; SPE, specificity; PPV, positive predictive value; NPV, negative predictive value; ACC, accuracy; Balanced ACC, balanced accuracy; LR, logistic regression; RF, random forest; NN, neural network; SVM, support vector machine; NB, naïve Bayes; DT, decision tree.

**Table 4:** Pairwise comparisons of area under the ROC curves (AUCs) between predictive models using DeLong’s test.

Model 1	Model 2	DeLong Z statistic	Bonferroni-adjusted P-value
**Logistic Regression**	**Random Forest**	3.25	0.017
**Logistic Regression**	**SVM Radial**	1.50	1
**Logistic Regression**	**Decision Tree**	4.22	<0.001
**Logistic Regression**	**Naive Bayes**	4.28	<0.001
**Logistic Regression**	**Neural Network**	−0.03	1
**Random Forest**	**SVM Radial**	−2.97	0.043
**Random Forest**	**Decision Tree**	2.49	0.187
**Random Forest**	**Naive Bayes**	1.43	1
**Random Forest**	**Neural Network**	−3.23	0.018
**SVM Radial**	**Decision Tree**	3.92	0.001
**SVM Radial**	**Naive Bayes**	3.29	0.015
**SVM Radial**	**Neural Network**	−1.59	1
**Decision Tree**	**Naive Bayes**	−0.98	1
**Decision Tree**	**Neural Network**	−4.22	<0.001
**Naive Bayes**	**Neural Network**	−4.22	<0.001

**Fig 1:**
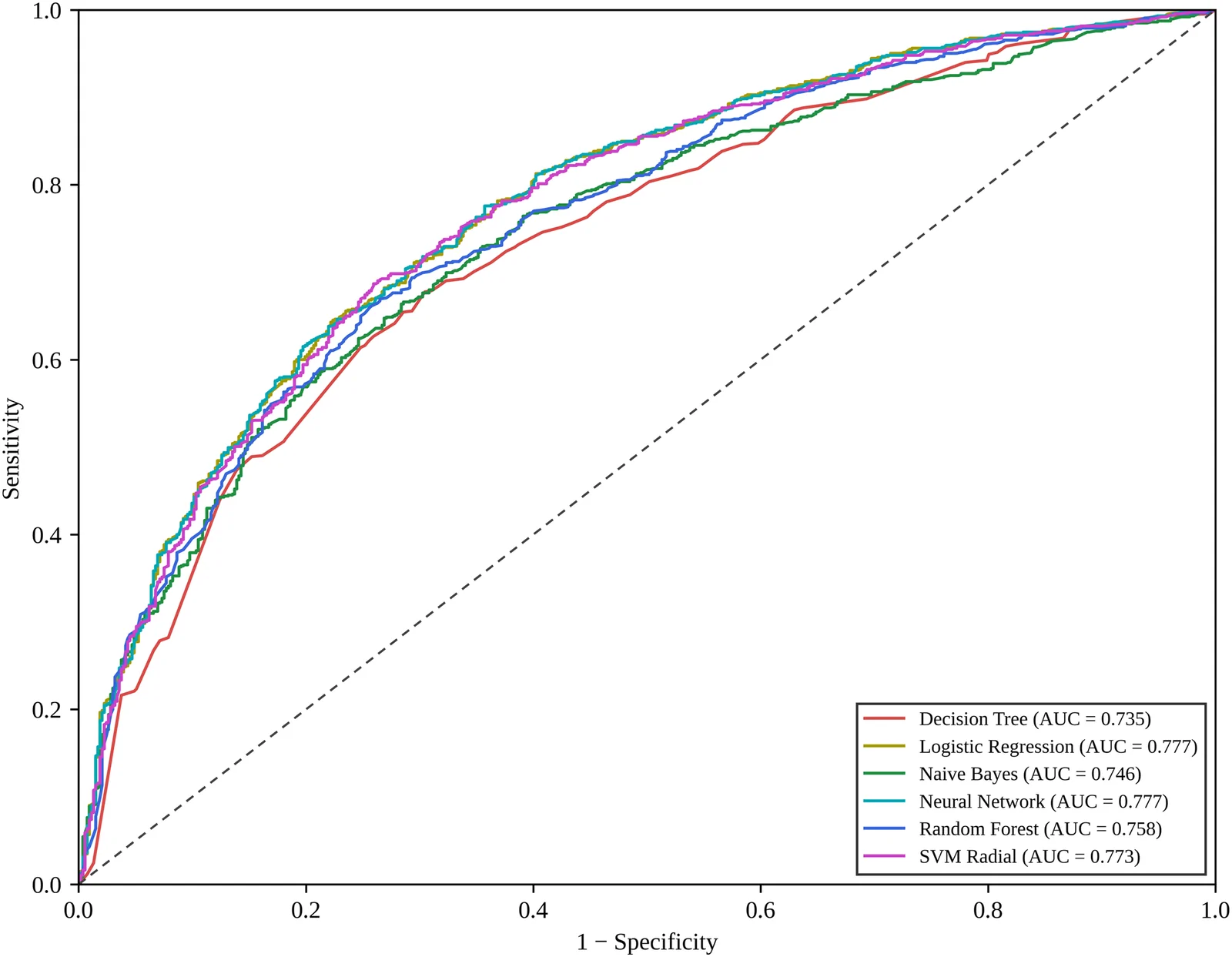
Estimated ROC curves based on different classification models.

Calibration patterns varied across models, with differences in the degree of agreement between predicted probabilities and observed food insecurity proportions. Relatively closer agreement with the reference diagonal line was observed for logistic regression, neural network, and support vector machine models, whereas higher deviations from the diagonal line were observed for the naïve Bayes model (Fig 2). These findings were generally consistent with the corresponding Brier score values reported in Table 3.

**Fig 2:**
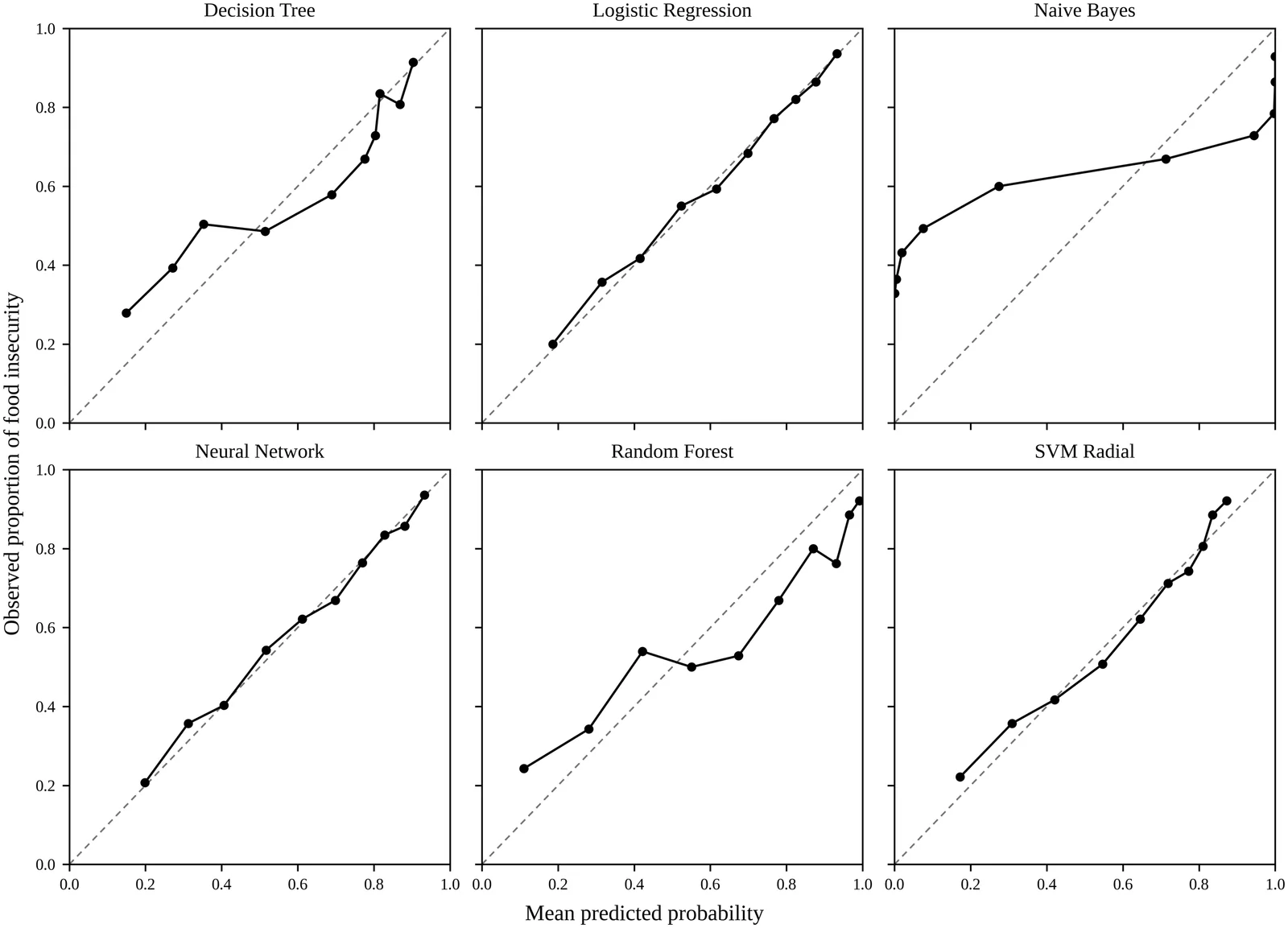
Calibration plots of logistic regression and machine learning models for predicting household food insecurity.

Table 5 compares predictor importance rankings obtained from the logistic regression (fitted using the forward selection method) and random forest models. Overall, a relatively similar ranking pattern was observed across the two modeling approaches for most important predictors. Household income was ranked as the most important predictor in both logistic regression and random forest models. Education level, car ownership, ethnicity, occupational status, and insurance-related variables also showed relatively similar importance rankings across these methods. Also, moderate differences in ranking were observed for some predictors, including house ownership and number of employed household members. Rank correlation analysis demonstrated a positive association between predictor rankings derived from logistic regression and random forest models. The Spearman rank correlation coefficient was 0.678 (P = 0.015), indicating significant agreement between the two modeling approaches regarding predictor importance ordering.

**Table 5:** Comparison of predictor importance rankings between logistic regression and random forest models.

Predictor	Logistic regression importance rank	Random forest importance rank	Rank consistency
**Income**	1	1	High consistency
**Education level**	2	3	High consistency
**Ethnicity**	3	5	High consistency
**Car ownership**	4	4	High consistency
**House ownership**	5	8	Moderate consistency
**Supplementary insurance**	6	6	High consistency
**Number of employed household members**	7	2	Moderate consistency
**Occupational status**	8	7	High consistency
**Insurance**	9	9	High consistency
**Number of household members**	10	10	High consistency
**Supporting organizations coverage**	11	11	High consistency
**Having incurable disease**	12	12	High consistency

## Discussion

This cross-sectional study, carried out between December 2021 and September 2022, evaluated the prevalence and risk indicators of FI among 4,948 households in Golestan Province, Iran. Our findings indicated a remarkable prevalence of some degree of FI (about 62% of households), with about one-third experiencing mild, 20.3% moderate, and 9.1% severe FI. This prevalence represents a 4.9% increase compared to 2016, likely driven by economic and environmental shocks, including the COVID-19 pandemic and regional flooding [7]. The findings therefore suggest that food insecurity continues to represent an important public health concern in northern Iran. Based on the present findings, similar to previous studies, several policy-related implications may be considered. The consistent identification of income, employment, and education suggests that these indicators may help inform screening and monitoring strategies for identifying households vulnerable to FI. Targeted economic support, employment assistance programs, educational interventions, and social protection policies directed toward socioeconomically vulnerable and female-headed households may contribute to reducing the burden of FI in similar populations [12,34]. Compared to developed countries such as Canada (14–17%) and Australia (26%), and developing countries including Nigeria (three sites with prevalence rates of 64.0%, 68.8% and 61.5%), Kenya (two sites with prevalence rates of 41.0% and 14.5%), Pakistan (41.1%), and Bangladesh (9.1%), the situation in Golestan reflects a critical food insecurity context [35–37].

In the present study, compared several conventional statistical and machine learning approaches for classifying household food insecurity using a large epidemiologic dataset composed primarily of categorical socioeconomic predictors. Overall, the predictive performances of logistic regression, neural network, and support vector machine models were relatively similar, whereas decision tree and naïve Bayes models generally showed lower discrimination and calibration performance.

Key determinants identified in this study—low household income, poor employment status, and low educational attainment of the household head—align with findings from previous national and international studies, which consistently highlight income and employment as primary predictors of FI [14,38–49]. Apparently, households with limited financial resources face restricted access to diverse and adequate food and are more vulnerable to economic shocks.

According to our findings, female-headed households were consistently at higher risk of FI. These findings are consistent with the obtained results from studies in Shiraz, Arak, Gilan, and other Iranian provinces, as well as international studies from Ethiopia, South Africa, and Brazil [38,39,44–49]. This disparity is largely attributable to limited employment opportunities, lower wages, and greater economic vulnerability.

In our study, it was shown that educational level of head of household was one of top five indicators of FI. This finding is in agreement with results from other studies like. It seems that this relationship can be described as two aspects; first, in countries like Iran higher levels of education is directly associated with higher income which results in better access to appropriate nutrition. Second, educated people have higher levels of knowledge, attitude and practice in consuming proper nutrients and consequently have lower risk of FI [14,49,50].

Employment status of the household head was another critical factor, corroborating previous findings from studies in Iran and other parts of the world [14,36,40,50,51]. Stable employment allows households to access a variety of nutritious foods and buffer against economic shocks.

According to our results, geographic location and ethnicity showed significant relationship with the risk of FI. Households in less developed areas or with heterogeneous migrant populations exhibited higher incidence of FI. Eastern regions of Golestan province, such as Minudasht, displayed moderate FI, whereas western regions, such as Bandar Torkaman, were comparatively better. These observations align with global evidence on the role of geographic and demographic factors in household food security [36,39,46]. Fars ethnicity appeared protective, consistent with Asadi-Lari et al. [52], whereas certain ethnic groups internationally have shown elevated FI risk [37,43,47].

Environmental and social shocks, including the COVID-19 pandemic and regional flooding, significantly influenced household FI, exacerbating economic pressures, limiting access to food resources, and increasing vulnerability among low-income households. These effects are in line with findings from other research across various settings [7,35,39].

Collectively, these results have substantial implications for policy and intervention strategies. Reducing FI prevalence necessitates a focus on low-income households, enhancement of education and employment opportunities, targeted support for female-headed households, and management of natural and social shocks. Integration of statistical analyses with machine learning models enables more precise identification of vulnerable households and informs the development of effective, evidence-based interventions. Overall, FI is a multidimensional and complex phenomenon influenced by socioeconomic, demographic, and environmental factors, necessitating comprehensive, evidence-based, and targeted strategies.

The findings from this study in Golestan Province, which reveal a food insecurity prevalence of 61.7%—marking a 4.9% increase since 2016—carry critical strategic implications for public health and socioeconomic policy. Our analysis identifies several key determinants of food insecurity, specifically low income, unemployment, lower educational attainment of the household head, female-headed status, lack of health insurance, and the presence of chronic or life-threatening illnesses. Furthermore, Logistic regression demonstrated predictive performance comparable to neural network and support vector machine models while maintaining greater interpretability.

Regarding resource allocation, evidence-based interventions should prioritize sustainable employment creation, targeted subsidies, and economic empowerment initiatives. Our data highlight specific vulnerability profiles that warrant immediate policy attention. Households with the highest risk of food insecurity include those with critically low income (85.6%), beneficiaries of welfare organizations (84.6%), households with an unemployed head (82.1%), those lacking basic insurance (79.6%), households managing life-threatening illnesses (75.1%), and female-headed households (70.9%).

We propose the integration of social screening protocols within the primary healthcare system, focusing on key socioeconomic markers such as income, insurance status, and employment stability. For patients diagnosed with chronic diseases, the implementation of specialized nutritional support packages could significantly improve health outcomes. Furthermore, we advocate for the development of an online food insecurity surveillance dashboard like SAMAT that leverages administrative datasets. Such a system would facilitate the precise targeting of social safety nets—including electronic food vouchers or conditional cash transfers—thereby mitigating resource leakage. Finally, these analytical models offer a robust framework for assessing the socioeconomic impacts of systemic shocks, such as natural disasters, pandemics, and inflationary pressures, providing policymakers with a dynamic tool for proactive intervention.

### Policy implications

The findings should be interpreted cautiously in light of the observational design of the study. Nevertheless, the identified socioeconomic predictors may help policymakers and public health practitioners better recognize groups at elevated risk of food insecurity. Interventions aimed at improving economic stability, educational opportunities, and employment conditions may contribute to reducing vulnerability to food insecurity. However, further research is required to evaluate the effectiveness of specific policy interventions and to assess the generalizability of the predictive models in other populations.

### Limitations

An important limitation of the present study is the absence of assessing external validation using an independent dataset from another population or geographic region. Although model performance was evaluated using an independent hold-out test set, this procedure represents internal validation rather than true external validation because both datasets originated from the same source population. Unfortunately, we did not have access to similar Iranian datasets to investigate the external validity of the fitted models. Consequently, the generalizability of the developed models to other geographic regions, populations, or time periods remains uncertain. Future studies should externally validate these models using independent datasets collected in different settings.

## Conclusion

In this population-based study, our findings showed that food insecurity affects a substantial proportion of households in Golestan Province. Food insecurity was associated with several socioeconomic characteristics, including household income, educational attainment, employment-related factors, ethnicity, and indicators of household economic resources. Collectively, the findings emphasize the importance of socioeconomic conditions in shaping household food insecurity and suggest that predictive modeling approaches may provide useful complementary tools for identifying households at elevated risk of food insecurity.

## Supporting information

S1 Table Definitions, hypothesized associations, and expected directions of the explanatory variables included in the analysis. (DOCX)

S2 Table Calibration performance of prediction models in the independent test dataset. (DOCX)
