# Supplementary material for: Food insecurity and its related indicators in an Iranian population: An application of machine learning methods in data from Golestan Province
Source: PLoS One. 2026 Oct 30;21(10):e0361131. doi: 10.1371/journal.pone.0361131 (PMC13722216; doi:10.1371/journal.pone.0361131)
Supplement: Supplementary file 1 [file pone.0361131.s001.docx]

| **Supplementary table 1.** Definitions, hypothesized associations, and expected directions of the explanatory variables included in the analysis | | | |
| --- | --- | --- | --- |
| **Variable** | **Definition** | **Hypothesis** | **Direction** |
| **place of residence** | It means being urban or rural. | It is assumed that residential location is related to food insecurity. | Indirect impact |
|  |  |  |  |
| **Age of the head of household** | This refers to the age of the head of the household. | As the age of the household head increases, the likelihood of reduced working capacity rises, thereby increasing the likelihood of food insecurity. | Indirect impact |
|  |  |  |  |
| **Gender of the head of household** | Gender of the head of household | Women have fewer job opportunities and earn less than men, making female-headed households more likely to be food insecure. | Direct impact |
|  |  |  |  |
| **Marital Status of the head of household** | Marital status of the head of household | Widowed and divorced people are more likely to be food insecure. | Indirect impact |
|  |  |  |  |
| **Ethnicity of the head of household** | Ethnicity of the head of household | Poverty and deprivation are more prevalent in certain ethnic groups. Among these groups, women are further disadvantaged in terms of employment, and any income they earn does not belong to them. | Indirect impact |
|  |  |  |  |
| **Number of household members** | Number of people living in a family | Large households are more vulnerable to food insecurity. | Direct impact |
|  |  |  |  |
| **Number of employed person in household** | Number of employed person in a household | The more people working in a household, the lower the likelihood of food insecurity. | Direct impact |
|  |  |  |  |
| **Education level of the head of household** | Years of education of the head of household | The higher the level of education of the head of household, the lower the likelihood of food insecurity. | Direct impact |
|  |  |  |  |
| **Employment status of the head of household** | Employment status of the head of household | Employment of the head of household reduces the likelihood of food insecurity | Direct impact |
|  |  |  |  |
| **Insurance status** | Having or not having health insurance | People who have health insurance are less likely to be food insecure. | Indirect impact |
|  |  |  |  |
| **Supplementary insurance** | Having or not having supplementary health insurance | Generally, people with higher economic levels have supplementary insurance and are less likely to be food insecure. | Indirect impact |
|  |  |  |  |
| **Cash subsidy** | Household receiving cash subsidy | Households with poor socioeconomic status generally receive cash subsidies, so they are more likely to be food insecure. | Indirect impact |
|  |  |  |  |
| **Under cover of supporting organizations** | Households covered by support institutions. | Households with low socioeconomic status are typically supported by welfare or aid institutions; however, they still face a higher risk of food insecurity. | Indirect impact |

| **Supplementary table 1 (Continued).** Definitions, hypothesized associations, and expected directions of the explanatory variables included in the analysis | | | |
| --- | --- | --- | --- |
| **Variable** | **Definition** | **Hypothesis** | **Direction** |
| **Having incurable disease** | A family member suffering from a difficult-to-treat disease | If any member of the household suffers from difficult-to-treat diseases, especially the breadwinner, it leads to a loss of household income. | Direct impact |
|  |  |  |  |
| **Having COVID-19** | Getting infected with Covid-19 | Getting COVID would result in a loss of household income for a period of time. | Indirect impact |
|  |  |  |  |
| **Car ownership** | Car ownership by each household member | Car ownership is a sign of good socioeconomic status. | Indirect impact |
|  |  |  |  |
| **House ownership** | House ownership by each household member | hause ownership is a sign of good socioeconomic status. | Indirect impact |
|  |  |  |  |
| **Income** | The average monthly income a person earns | The lower a person's monthly income, the greater the likelihood of food insecurity. | Direct impact |
