# Supplementary material for: Food insecurity and its related indicators in an Iranian population: An application of machine learning methods in data from Golestan Province
Source: PLoS One. 2026 Oct 30;21(10):e0361131. doi: 10.1371/journal.pone.0361131 (PMC13722216; doi:10.1371/journal.pone.0361131)
Supplement: Supplementary file 2 [file pone.0361131.s002.docx]

| **Supplementary table 2.** Calibration performance of prediction models in the independent test dataset | | |
| --- | --- | --- |
| **Model** | **Intercept**  **(95% CI)** | **Slope**  **(95% CI)** |
| Neural network | 0.042 (-0.086, 0.171) | 0.935 (0.819, 1.050) |
|  |  |  |
| Logistic Regression | 0.044 (-0.885, 0.174) | 0.934 (0.818, 1.050) |
|  |  |  |
| Support vector machine | 0.062 (-0.068, 0.194) | 0.965 (0.844, 1.090) |
|  |  |  |
| Random Forest | 0.038 (-0.089, 0.167) | 0.483 (0.413, 0.552) |
|  |  |  |
| Naïve Bayes | 0.407 (0.289, 0.526) | 0.151 (0.130, 0.173) |
|  |  |  |
| Decision Tree | 0.218 (0.096, 0.339) | 0.550 (0.458, 0.642) |
