# Supplementary material for: Food insecurity and its related indicators in an Iranian population: An application of machine learning methods in data from Golestan Province
Source: PLoS One. 2026 Oct 30;21(10):e0361131. doi: 10.1371/journal.pone.0361131 (PMC13722216; doi:10.1371/journal.pone.0361131)
Supplement: Supplementary file 3 [file pone.0361131.s004.docx]

Manuscript ID: PONE-D-26-09587

Dear Academic Editor of PLoS ONE,

At first, we should express our special thanks to you and the reviewers for the valuable and helpful comments. Hereby, it should be mentioned that we read the comments very carefully and made substantial changes in the text.

Following the reviewers’ suggestions, the analytical pipeline was substantially revised to improve methodological rigor and reduce the risk of overfitting. Specifically, the revised analysis incorporated repeated cross-validation within the training dataset, independent test-set evaluation, transparent hyperparameter tuning, calibration assessment, Brier score evaluation, and a pre-specified prevalence-based classification threshold. After implementation of these stricter validation procedures, the relative performance of the models changed slightly compared with the initial submission. In the revised analyses, logistic regression and neural network models demonstrated marginally better overall discrimination and calibration performance than the random forest model on the independent test dataset. This finding is methodologically plausible given the characteristics of the present dataset, which primarily consisted of categorical socioeconomic predictors with relatively stable and interpretable associations with food insecurity. In such settings, simpler and more parsimonious models may generalize as well as or better than more flexible ensemble approaches. Importantly, the revised results emphasize that more complex machine learning algorithms do not necessarily outperform conventional statistical models in all epidemiologic prediction settings.

The manuscript has been revised accordingly to avoid overstating the superiority of any individual machine learning approach.

**Response to the Editor's comments:**

**Comment 1:** Please ensure that your manuscript meets PLOS ONE's style requirements, including those for file naming.

**Response:**

The revision has been made according to the editor's comment.

**Comment 2:** Please include your tables as part of your main manuscript and remove the individual files. Please note that supplementary tables (should remain/ be uploaded) as separate "supporting information" files.

**Response:**

All tables were included in the text according to the editor's comment.

**Comment 3:** We note that you have indicated that there are restrictions to data sharing for this study. For studies involving human research participant data or other sensitive data, we encourage authors to share de-identified or anonymized data. However, when data cannot be publicly shared for ethical reasons, we allow authors to make their data sets available upon request.

**Response:**

The data is now available upon request (line 480-483).

**Comment 4:** Ethics statement should only appear in Methods.

**Response:**

The ethics statement has been moved to the Methods section according to the editor's comment (line 257-261).

**Response to reviewers #1:**

**Comment 1:** Similar studies applying ML to food insecurity prediction already exist in public health literature.

**Response:**

Thank you for this valuable comment. We agree that previous studies have applied machine learning (ML) methods for food insecurity (FI) prediction and classification in different public health settings. However, the primary contribution of the current study is not merely the application of ML techniques, but rather the comparative evaluation of conventional statistical approaches and multiple ML algorithms within a large population-based dataset from northern Iran. Previous Iranian studies have mainly focused on identifying determinants of FI using conventional statistical methods, particularly logistic regression models and cross-sectional analytical approaches (1,2). In contrast, evidence regarding the simultaneous application and comparison of ML and traditional statistical approaches for identifying FI risk indicators and classifying households remains limited in the Iranian population.

To address the reviewer’s concern and avoid overstating the novelty of the study, we revised the Introduction section to better position the present work within the existing literature and clarified that the study contributes additional evidence regarding the applicability and comparative performance of different analytical approaches for FI risk assessment and classification in an Iranian population-based setting.

1. Honarvar MR, Gholami M, Abdollahi Z, Ghotbabadi FS, Lashkarboluki F, Najafzadeh M, et al. Household food insecurity and associated factors in the Northeast of Iran: a cross-sectional study: Household food security in Northern Iran. BMC nutrition. 2023;9(1):5.
2. Khodabakhshzadeh S, Moradabadi SA, Khodabakhshzadeh S, Baniasadi M, Khalilabad TH. Food security and its related factors in the rural households of Bam city in Iran: a logistic regression model approach. Archives of Iranian medicine. 2018;21(1):26-8.

**Comment 2:** The manuscript mainly confirms known socioeconomic determinants (income, education, employment).

**Response:**

We thank the reviewer for this valuable comment. The primary aim of the present study was not to establish novel socioeconomic determinants of FI, but rather to evaluate and compare the performance of conventional statistical approaches and machine learning algorithms for identifying and classifying FI risk factors within a large population-based dataset from northern Iran.

**Comment 3:** ML is used primarily for prediction rather than generating new theoretical insights.

**Response:**

We thank the reviewer for this important conceptual comment. We revised the manuscript according to the reviewer's comment.

**Comment 4:** Moderate originality, incremental contribution rather than innovative advancement.

**Response:**

We thank the reviewer for this thoughtful assessment. In this manuscript, we did not aim to present an innovative advancement in this field. Instead, our main aims were to use a large sample data for identifying some of the most important indicators of food insecurity and employ these indicators to present a framework based on both the classic and ML methodologies for predicting FI in Iranian population.

**Comment 5:** The manuscript does not sufficiently emphasize the limitation of causal inference.

**Response:**

We thank the reviewer for highlighting this important methodological point. We agree that, given the cross-sectional design of the study, causal relationships cannot be inferred from the observed associations. The findings should therefore be interpreted as associations rather than causal effects.

**Comment 6:** Sampling strategy is insufficiently described (randomization, clustering, weighting).

**Response:**

We thank the reviewer for highlighting the need for a clearer description of the sampling strategy. The Study design section has been revised according to the reviewer's comment (Lines 127–146 & 180–186).

**Comment 7:** Representativeness of Golestan Province relative to Iran is not justified.

**Response:**

We thank the reviewer for this important comment regarding the generalizability of our findings. We acknowledge that Golestan Province may not be fully representative of the entire Iranian population, given the country’s geographic, socioeconomic, and cultural diversity. However, Golestan Province is characterized by substantial heterogeneity in terms of ethnicity, socioeconomic status, and living conditions, which makes it a fairly representative sample of all Iranians and valuable setting for studying food insecurity in a diverse population.

**Comment 8:** No discussion of cultural validation or reliability testing within this dataset.

**Response:**

We thank the reviewer for this important comment regarding the validity and reliability of the measurement tool. Additional information regarding the validity and reliability of the HFIAS has been added to the Methods section (Lines 149–152).

**Comment 9:** Machine Learning Methodology.

This is the most critical methodological weakness.

**Response:**

We thank the reviewer for this important and constructive comment. We have revised the manuscript to provide a transparent and reproducible machine learning workflow (Lines 191–256).

**Comment 10:** Missing or unclear details:

Data preprocessing steps.

Handling of missing data.

Feature scaling or normalization.

Hyperparameter tuning strategy.

Overfitting prevention measures.

Without these details, reproducibility is limited.

**Response:**

We thank the reviewer for this important and constructive comment. The Statistical Analysis section has been extensively revised.

**Comment 11:** Accuracy alone is insufficient for imbalanced datasets.

**Response:**

Thank you for this comment. In addition to accuracy, we report sensitivity, specificity, positive predictive value (PPV), negative predictive value (NPV), balanced accuracy, area under the receiver operating characteristic curve (AUC) with 95% confidence intervals, and Brier score for all machine learning models. We also added calibration plots to evaluate agreement between predicted probabilities and observed event rates.

Furthermore, stratified sampling was used during both train–test splitting and repeated cross-validation procedures to preserve the original class distribution across datasets. Because the degree of class imbalance was not extreme, and to maintain the real-world distribution of food insecurity outcomes, no artificial class balancing techniques were applied.

**Comment 12:** No calibration metrics (Brier score, calibration plots).

**Response:**

We thank the reviewer for this valuable comment. We revised the manuscript according to the reviewer's comment.

**Comment 13:** No external validation dataset.

**Response:**

We thank the reviewer for this important comment. The authors are aware of the importance of assessing the external validity of the methods. However, lack of access to similar data in our country makes it impossible to evaluate the external validity of the utilized models in other datasets. We mentioned this limitation in the discussion section (line 444–450).

**Comment 14:** Interpretation sometimes implies explanatory power rather than predictive association.

**Response:**

We thank the reviewer for this important comment. We revised the text according to the reviewer's comment.

**Comment 15:** No comparison of effect sizes between ML and regression outputs.

**Response:**

We thank the reviewer for this insightful comment. Because most machine learning algorithms do not produce directly interpretable effect size estimates comparable to odds ratios, predictor importance measures derived from machine learning models were compared qualitatively with adjusted odds ratios obtained from logistic regression.

Most machine learning algorithms used in the present study are prediction-oriented rather than inference-oriented and therefore do not generate directly interpretable effect size estimates comparable to odds ratios derived from logistic regression models. To address the reviewer’s concern regarding variable influence across modeling approaches, we compared adjusted odds ratios from multivariable logistic regression with model-specific variable importance measures obtained from machine learning algorithms. This comparison was intended to evaluate the consistency of predictor relevance across conventional statistical modeling and machine learning frameworks rather than to establish direct equivalence between odds ratios and machine learning importance metrics.

**Comment 16:** Lack of practical policy translation.

**Response:**

We thank the reviewer for this valuable comment. The Discussion section have been expanded to include more explicit and actionable policy implications.

**Comment 17:** Confusion between prediction and inference. ML models predict outcomes but do not establish causality.

**Response:**

We thank the reviewer for this important clarification. The Introduction, Methods, Results, and Discussion sections have been revised to clearly distinguish predictive modeling from causal inference and to avoid any implication of causality.

**Comment 18:** Claim of “superior performance”. Improvement over traditional methods appears modest and not statistically tested.

**Response:**

We thank the reviewer for this important comment. The authors confirm that the predictive performance of the employed models are very close and the differences between estimated indices are rather insignificant. In the original manuscript, the term “superior performance” may have overstated the observed differences. We have revised the manuscript to use more cautious and accurate language, such as "slightly higher", when describing model results. We added this explanation & Delong's test in the text.

**Comment 19:** No uncertainty quantification for ML variable importance. Importance rankings may be unstable.

**Response:**

We thank the reviewer for this important methodological comment. In the revised manuscript, we expanded the analysis to address uncertainty and stability of predictor importance estimates. Specifically, for the logistic regression model, bootstrap resampling (200 iterations) was performed to estimate selection frequency, mean importance values, and bootstrap-based for predictors. This approach was incorporated to evaluate the robustness and stability of variable importance rankings across repeated samples. In addition, the revised analyses demonstrated that conventional logistic regression achieved predictive performance comparable to several machine-learning models, with only modest differences in discrimination metrics.

**Response to reviewers #2:**

**Abstract**

**Comment 1:** The abstract should present the key econometric analysis results more clearly and precisely to enhance its attractiveness and informativeness for readers.

**Response:**

We thank the reviewer for this insightful comment.

**Introduction**

**Comment 2:** In paragraph 2, the statement: “According to reports in 2023, approximately two billion people (about 28.9% of the world's population) experience moderate to severe food insecurity” requires proper citation. Please clearly indicate the source of this data to ensure its validity.

**Response:**

We thank the reviewer for this insightful comment. We have now added an appropriate citation to support the global food insecurity estimates reported in the Introduction.

**Comment 3:** A similar issue appears in paragraph 4, where claims are made without appropriate references.

**Response:**

We thank the reviewer for this insightful comment. We carefully reviewed paragraph 4 and sentences that required references.

**Comment 4:** In paragraph 5, you mention that “various studies” have identified key determinants of food insecurity, yet no specific studies are cited. At least a few representative and relevant references should be included to support this statement. Overall, the introduction should also highlight the methodological novelty of the study and clearly distinguish it from previous research.

**Response:**

We thank the reviewer for this insightful comment.

**Methods**
**Comment 5:** In the methodology section, it is recommended to include the sample size determination method along with the relevant formula to improve transparency and reproducibility. Additionally, a clear and concise description of the study area is essential and should be included.

**Response:**

We thank the reviewer for this insightful comment (Line 131).

**Variables Used**

**Comment 6:** It would be helpful to present all variables in a table, including their definitions and expected signs (hypotheses), to improve clarity and readability.

**Response:**

We thank the reviewer for this insightful comment.

**Data Analysis**

**Comment 7:** The section titled “Statistical Analysis” includes econometric methods such as univariate and multivariate logistic regression models. Therefore, the title should be revised to accurately reflect the econometric nature of the analysis.

**Response:**

We thank the reviewer for this insightful comment.

**Results and Discussion**

**Comment 8:** While the results are presented and interpreted, they should be supported by comparisons with existing literature. Engaging with other studies will strengthen the discussion and enhance the credibility of the findings. The discussion section should go beyond description and provide deeper analytical insights, clearly articulating the scientific and policy implications of the findings.

**Response:**

We thank the reviewer for this insightful comment.

**Conclusion**
**Comment 9:** The conclusion should clearly present the core message of the study and provide a concise synthesis of the main findings. It should not be combined with recommendations. Please present the conclusion in paragraph form and avoid bullet points.

Recommendations
The recommendations should be specific and actionable, clearly indicating what should be done, how it can be implemented, and which stakeholders are responsible for each action.

**Response:**

We thank the reviewer for this insightful comment.

**References**
**Comment 10:** The referencing style does not follow the journal guidelines (PLOS ONE). In-text citations should be presented using numbered brackets, whereas the current manuscript uses parenthetical citations. This should be corrected to align with the journal’s requirements.

**Response:**

We thank the reviewer for this insightful comment.

**Response to reviewers #3:**

**Comment 1:** Its relevance and overarching goal as contributed to the scientific community has to be clearly laid out to match the scope of PLOS one, which has a broad readership.

**Response:**

We thank the reviewer for this insightful comment. To match the scope of journal, we made some changes and corrections in the text.

**Comment 2:** Please consider a justification for the use of machine-learning methods beyond logistic regression. The predictive gain over traditional modeling appears modest, so the authors should explain why ML adds meaningful value in this dataset.

**Response:**

We thank the reviewer for this insightful comment. Comparing the results of traditional and machine learning methods in predicting food insecurity was one of the main objectives in the proposal of the current study. We aimed to investigate and compare the predictive performance of classic and ML methods in this data set. Since this proposal is part of Ph.D thesis in department of biostatistics, in the next phases we aim to develop innovative machine learning approaches for the analysis of similar data sets considering both the main and interaction effects of predictors in the modeling process.

**Comment 3:** Methods section requires significant improvement. The analysis workflow for the machine-learning models is insufficiently described. The authors should report train/test partitioning, cross-validation strategy, hyperparameter tuning, handling of class imbalance, and whether performance metrics were evaluated on independent data. Overall for clarity the programs used for this work and their licenses have to be clearly stated. Please refer to relevant literature.

**Response:**

We thank the reviewer for this insightful comment. We substantially revised the Methods section according to the reviewer's comment.

**Comment 4:** I am skeptical about the authors over interpreting their cross-sectional study. Abbreviation jargons must be decoded in the tables and figures’ legends. For example Table 3. including HFIAS, RF, NN, SVM, NB, DT, PPV, NPV, AUC, and ACC.

**Response:**

We thank the reviewer for this insightful comment. We carefully revised the manuscript to avoid over interpretation of findings from this cross-sectional study and to maintain a predictive rather than causal interpretation throughout the manuscript. Specifically, statements implying superiority or causal inference were moderated and replaced with more cautious language emphasizing comparative predictive performance. In addition, all abbreviations used in tables and figure legends have now been fully defined for clarity and readability.

**Comment 5:** The model-selection strategy needs more rigor. The use of univariate screening followed by multivariable modeling may introduce bias, and the rationale for selecting predictors should be clarified or strengthened with theory-driven variable selection.

**Response:**

We thank the reviewer for this insightful comment. The multivariable analysis was restructured using forward stepwise logistic regression based on the Akaike Information Criterion (AIC), rather than relying solely on univariate statistical significance for selecting the predictors.

**Comment 6:** Figure 1 Y axis – what does 1-specifity term mean?

**Response:**

We thank the reviewer for this insightful comment. In receiver operating characteristic (ROC) analysis, the term “1-specificity” refers to the false positive rate, which represents the proportion of non-events incorrectly classified as events by the prediction model. In the context of the present study, it indicates the proportion of food-secure households incorrectly classified as food insecure. The use of sensitivity on the y-axis and 1-specificity on the x-axis is the conventional and standard presentation of ROC curves in diagnostic and predictive modeling studies. We retained this terminology to maintain consistency with established ROC methodology.

**Comment 7:** The manuscript should address clustering from the multistage sampling design. If clustering was not incorporated into the regression analysis, standard errors and p-values may be inaccurate.

**Response:**

We thank the reviewer for this insightful comment. We reanalyzed the data to incorporate the weighting factors in the model fitting process. The process was explained in the Methods section.

**Comment 8:** Where is the random forest variable results described? I solicit a thorough and rigorous analyses of all the results the authors submitted for evaluation in the form of figures and tables. The results section was carelessly done.

**Response:**

We thank the reviewer for this insightful comment. In table 5, we added some descriptions about the output of random forest analysis including variable importance rankings and reporting the correlation between these ranks with those obtained from the multivariable logistic regression model.

**Comment 9:** Data availability statements and relevant consent/IRB statements are missing. Again, please refer to relevant epidemiology references to format the manuscript.

**Response:**

We thank the reviewer for this insightful comment. We added the data availability statement in the text.

**Comment 10:** Please check all tables and figures, update legends and check consistency.

**Response:**

We thank the reviewer for this insightful comment. We rechecked the tables and figures.

**Comment 11:** In Statistical Analysis section of methods line 155 – why p<0.1 was considered?

**Response:**

We thank the reviewer for this insightful comment. We included the results from fitting the multivariable logistic regression with forward selection method instead of screening the variables using the univariate analysis.
