# Supplementary material for: Food insecurity and its related indicators in an Iranian population: An application of machine learning methods in data from Golestan Province
Source: PLoS One. 2026 Oct 30;21(10):e0361131. doi: 10.1371/journal.pone.0361131 (PMC13722216; doi:10.1371/journal.pone.0361131)
Supplement: Supplementary file 4 [file pone.0361131.s005.docx]

Manuscript ID: PONE-D-26-09587R1

Dear Academic Editor of PLoS ONE,

We sincerely appreciate your careful review of our manuscript and your constructive and insightful comments. We are grateful for the opportunity to further improve the manuscript in response to your recommendations. We have carefully considered each comment and have revised the manuscript accordingly. A detailed, point-by-point response to the Editor's comments is provided below.

**Response to the Editor's comments:**

**Comment 1:** The manuscript states that missing observations were handled through complete-case analysis because the missing rate was below 6%. However, the mechanism of missingness (MCAR, MAR, or potentially MNAR) is not sufficiently demonstrated.

**Response:**

We thank the Academic Editor for this important observation. In the revised manuscript, we have expanded the description of missing-data handling. Prior to conducting complete-case analysis, we examined the pattern and extent of missingness across variables. The overall proportion of missing data was low (below 6% for all variables), and no clear evidence of systematic differences in missingness across the primary outcome categories or key demographic characteristics was observed. Given the limited amount of missing information and the absence of evidence suggesting substantial informative missingness, complete-case analysis was retained as the primary approach. We have clarified this rationale in the Statistical Analysis section.

**Comment 2:** The manuscript uses a fixed threshold of 0.61 for all classification models because it approximately reflects the observed prevalence of food insecurity. However, prevalence matching does not necessarily establish that 0.61 is the optimal or clinically/policy-relevant threshold.

**Response:**

We appreciate this valuable comment. We agree that a prevalence-based threshold (fixed threshold of 0.61) does not necessarily represent the optimal decision threshold for clinical or policy applications. The primary objective of this study was to compare the predictive performance of different modeling approaches under a common evaluation framework rather than to develop a decision-support tool with a specific operational threshold. Therefore, a fixed threshold corresponding approximately to the observed prevalence of food insecurity was applied uniformly across all models to facilitate direct comparison of classification metrics. Importantly, the primary performance measure used for model comparison was based on the Youden index in ROC curve analysis, which is independent of any particular classification threshold.

**Comment 3:** The authors should consider reporting a quantitative calibration measure, such as calibration intercept and calibration slope, and explain which model demonstrates the best calibration. This is particularly important because the study emphasizes prediction rather than merely discrimination.

**Response:**

Thank you for this valuable suggestion. In the revised manuscript, we supplemented the calibration plots with quantitative calibration measures, including calibration intercepts and calibration slopes, calculated using the independent test dataset. The results have been added as Supplementary Table 2.

**Comment 4:** The authors should distinguish between internal validation through the 30% test set and genuine external validation in another geographic population/time period.

**Response:**

We thank the Academic Editor for highlighting this important issue. In the revised manuscript, we have clarified that the independent 30% test dataset represents an internal validation procedure because it was derived from the same source population as the training dataset. We explicitly distinguish this approach from external validation, which requires evaluation in an independent population, geographic region, or time period. We have revised the Methods, Results, and Limitations sections accordingly and emphasize that external validation was not performed in the present study.

**Comment 5:** The conclusion should focus on the principal empirical findings, while policy recommendations should be presented separately and expressed cautiously.

**Response:**

We appreciate this recommendation. In the revised manuscript, the Conclusion section has been rewritten to focus primarily on the main empirical findings of the study, including the prevalence of food insecurity, the identified socioeconomic predictors, and the comparative performance of the evaluated predictive models. Policy implications have been moved to the Discussion section and rephrased more cautiously to reflect their exploratory and contextual nature.
