# Supplementary material for: Food insecurity and its related indicators in an Iranian population: An application of machine learning methods in data from Golestan Province
Source: PLoS One. 2026 Oct 30;21(10):e0361131. doi: 10.1371/journal.pone.0361131 (PMC13722216; doi:10.1371/journal.pone.0361131)
Supplement: Peer review history

# Peer review: Decision Letter 0

**DOI:** 10.1371/journal.pone.0361131.r001

**Authors:** Jamil Afzal

27 Apr 2026

Dear Dr. Zayeri,

Thank you for submitting your manuscript to PLOS ONE. After careful consideration, we feel that it has merit but does not fully meet PLOS ONE’s publication criteria as it currently stands. Therefore, we invite you to submit a revised version of the manuscript that addresses the points raised during the review process.

Please submit your revised manuscript by Jun 11 2026 11:59PM. If you will need more time than this to complete your revisions, please reply to this message or contact the journal office at plosone@plos.org. When you're ready to submit your revision, log on to https://www.editorialmanager.com/pone/ and select the 'Submissions Needing Revision' folder to locate your manuscript file.

- A letter that responds to each point raised by the academic editor and reviewer(s). You should upload this letter as a separate file labeled 'Response to Reviewers'.
- A marked-up copy of your manuscript that highlights changes made to the original version. You should upload this as a separate file labeled 'Revised Manuscript with Track Changes'.
- An unmarked version of your revised paper without tracked changes. You should upload this as a separate file labeled 'Manuscript'.

If you would like to make changes to your financial disclosure, please include your updated statement in your cover letter. Guidelines for resubmitting your figure files are available below the reviewer comments at the end of this letter.

If applicable, we recommend that you deposit your laboratory protocols in protocols.io to enhance the reproducibility of your results. Protocols.io assigns your protocol its own identifier (DOI) so that it can be cited independently in the future. For instructions see: https://journals.plos.org/plosone/s/submission-guidelines#loc-laboratory-protocols. Additionally, PLOS ONE offers an option for publishing peer-reviewed Lab Protocol articles, which describe protocols hosted on protocols.io. Read more information on sharing protocols at https://plos.org/protocols?utm_medium=editorial-email&utm_source=authorletters&utm_campaign=protocols.

As the corresponding author, your ORCID iD is verified in the submission system and will appear in the published article. PLOS supports the use of ORCID, and we encourage all coauthors to register for an ORCID iD and use it as well. Please encourage your coauthors to verify their ORCID iD within the submission system before final acceptance, as unverified ORCID iDs will not appear in the published article. *Only*  the individual author can complete the verification step; PLOS staff *cannot*  verify ORCID iDs on behalf of authors.

We look forward to receiving your revised manuscript.

Kind regards,

Jamil Afzal, Ph.D, Post Doc

Academic Editor

PLOS One

Journal Requirements:

1. Please ensure that your manuscript meets PLOS ONE's style requirements, including those for file naming. The PLOS ONE style templates can be found at

https://journals.plos.org/plosone/s/file?id=wjVg/PLOSOne_formatting_sample_main_body.pdf and

https://journals.plos.org/plosone/s/file?id=ba62/PLOSOne_formatting_sample_title_authors_affiliations.pdf.

2. Please include your tables as part of your main manuscript and remove the individual files. Please note that supplementary tables (should remain/ be uploaded) as separate "supporting information" files.

3. We note that you have indicated that there are restrictions to data sharing for this study. For studies involving human research participant data or other sensitive data, we encourage authors to share de-identified or anonymized data. However, when data cannot be publicly shared for ethical reasons, we allow authors to make their data sets available upon request. For information on unacceptable data access restrictions, please see http://journals.plos.org/plosone/s/data-availability#loc-unacceptable-data-access-restrictions.

Before we proceed with your manuscript, please address the following prompts:

a) If there are ethical or legal restrictions on sharing a de-identified data set, please explain them in detail (e.g., data contain potentially identifying or sensitive patient information, data are owned by a third-party organization, etc.) and who has imposed them (e.g., a Research Ethics Committee or Institutional Review Board, etc.). Please also provide contact information for a data access committee, ethics committee, or other institutional body to which data requests may be sent.

b) If there are no restrictions, please upload the minimal anonymized data set necessary to replicate your study findings to a stable, public repository and provide us with the relevant URLs, DOIs, or accession numbers. Please see http://www.bmj.com/content/340/bmj.c181.long for guidelines on how to de-identify and prepare clinical data for publication. For a list of recommended repositories, please see https://journals.plos.org/plosone/s/recommended-repositories. You also have the option of uploading the data as Supporting Information files, but we would recommend depositing data directly to a data repository if possible.

Please update your Data Availability statement in the submission form accordingly.

4. Your ethics statement should only appear in the Methods section of your manuscript. If your ethics statement is written in any section besides the Methods, please move it to the Methods section and delete it from any other section. Please ensure that your ethics statement is included in your manuscript, as the ethics statement entered into the online submission form will not be published alongside your manuscript.

5. If the reviewer comments include a recommendation to cite specific previously published works, please review and evaluate these publications to determine whether they are relevant and should be cited. There is no requirement to cite these works unless the editor has indicated otherwise.

[Note: HTML markup is below. Please do not edit.]

Reviewers' comments:

Reviewer's Responses to Questions

**Comments to the Author**

1. Is the manuscript technically sound, and do the data support the conclusions?

Reviewer #1: Partly

Reviewer #2: Partly

Reviewer #3: Partly

**********

2. Has the statistical analysis been performed appropriately and rigorously?

Reviewer #1: Yes

Reviewer #2: Yes

Reviewer #3: N/A

**********

3. Have the authors made all data underlying the findings in their manuscript fully available?

The PLOS Data policy

Reviewer #1: Yes

Reviewer #2: Yes

Reviewer #3: No

**********

4. Is the manuscript presented in an intelligible fashion and written in standard English?

Reviewer #1: Yes

Reviewer #2: Yes

Reviewer #3: Yes

**********

Reviewer #1: -Similar studies applying ML to food insecurity prediction already exist in public health literature.

-The manuscript mainly confirms known socioeconomic determinants (income, education, employment).

-ML is used primarily for prediction rather than generating new theoretical insights.

-Moderate originality, incremental contribution rather than innovative advancement.

-The manuscript does not sufficiently emphasize the limitation of causal inference.

-Sampling strategy is insufficiently described (randomization, clustering, weighting).

-Representativeness of Golestan Province relative to Iran is not justified.

-No discussion of cultural validation or reliability testing within this dataset.

-Machine Learning Methodology

This is the most critical methodological weakness.

-Missing or unclear details:

Data preprocessing steps.

Handling of missing data.

Feature scaling or normalization.

Hyperparameter tuning strategy.

Overfitting prevention measures.

Without these details, reproducibility is limited.

-Accuracy alone is insufficient for imbalanced datasets.

-No calibration metrics (Brier score, calibration plots).

-No external validation dataset.

-Interpretation sometimes implies explanatory power rather than predictive association.

-No comparison of effect sizes between ML and regression outputs.

-Lack of practical policy translation.

-Confusion between prediction and inference

ML models predict outcomes but do not establish causality.

-Claim of “superior performance”

Improvement over traditional methods appears modest and not statistically tested.

-No uncertainty quantification for ML variable importance

Importance rankings may be unstable.

Reviewer #2: I have carefully reviewed the manuscript entitled “Food Insecurity and its Related Indicators in an Iranian Population: an application of machine learning methods in data from Golestan Province”. The study is timely and makes a valuable contribution to the field of food security research. However, several issues need to be addressed before the manuscript can be considered for publication. To improve the overall quality and clarity of the paper, I offer the following suggestions:

Abstract

The abstract should present the key econometric analysis results more clearly and precisely to enhance its attractiveness and informativeness for readers.

Introduction

In paragraph 2, the statement: “According to reports in 2023, approximately two billion people (about 28.9% of the world's population) experience moderate to severe food insecurity” requires proper citation. Please clearly indicate the source of this data to ensure its validity.

A similar issue appears in paragraph 4, where claims are made without appropriate references.

In paragraph 5, you mention that “various studies” have identified key determinants of food insecurity, yet no specific studies are cited. At least a few representative and relevant references should be included to support this statement. Overall, the introduction should also highlight the methodological novelty of the study and clearly distinguish it from previous research.

Methods

In the methodology section, it is recommended to include the sample size determination method along with the relevant formula to improve transparency and reproducibility. Additionally, a clear and concise description of the study area is essential and should be included.

Variables Used

It would be helpful to present all variables in a table, including their definitions and expected signs (hypotheses), to improve clarity and readability.

Data Analysis

The section titled “Statistical Analysis” includes econometric methods such as univariate and multivariate logistic regression models. Therefore, the title should be revised to accurately reflect the econometric nature of the analysis.

Results and Discussion

While the results are presented and interpreted, they should be supported by comparisons with existing literature. Engaging with other studies will strengthen the discussion and enhance the credibility of the findings. The discussion section should go beyond description and provide deeper analytical insights, clearly articulating the scientific and policy implications of the findings.

Conclusion

The conclusion should clearly present the core message of the study and provide a concise synthesis of the main findings. It should not be combined with recommendations. Please present the conclusion in paragraph form and avoid bullet points.

Recommendations

The recommendations should be specific and actionable, clearly indicating what should be done, how it can be implemented, and which stakeholders are responsible for each action.

References

The referencing style does not follow the journal guidelines (PLOS ONE). In-text citations should be presented using numbered brackets, whereas the current manuscript uses parenthetical citations. This should be corrected to align with the journal’s requirements.

Overall Assessment

The manuscript addresses an important topic and has the potential to contribute meaningfully to the field. With the above revisions, it could become suitable for publication.

Reviewer #3: The article Food insecurity and its Related Indicators in an Iranian Population: an application of machine learning methods in data from Golestan Province examines household food insecurity in Golestan Province, Iran, and compares conventional statistical modeling with several machine-learning approaches. I have several points that need to be addressed for proper understanding of this manuscript. Firstly, its relevance and overarching goal as contributed to the scientific community has to be clearly laid out to match the scope of PLOS one, which has a broad readership. The topic is important and the sample size is substantial, but the expectation of readers to use the current methodologies and its usefulness requires further clarification. The article is not adequately written fulfilling a baseline scientific standard, while there are definite scope for improvements. I request a major revision in this aspect and the authors might consider my following comments.

Please consider a justification for the use of machine-learning methods beyond logistic regression. The predictive gain over traditional modeling appears modest, so the authors should explain why ML adds meaningful value in this dataset.

Methods section requires significant improvement. The analysis workflow for the machine-learning models is insufficiently described. The authors should report train/test partitioning, cross-validation strategy, hyperparameter tuning, handling of class imbalance, and whether performance metrics were evaluated on independent data. Overall for clarity the programs used for this work and their licenses have to be clearly stated. Please refer to relevant literature.

I am skeptical about the authors overinterpreting their cross-sectional study. Abbreviation jargons must be decoded in the tables and figures’ legends. For example Table 3. including HFIAS, RF, NN, SVM, NB, DT, PPV, NPV, AUC, and ACC.

The model-selection strategy needs more rigor. The use of univariate screening followed by multivariable modeling may introduce bias, and the rationale for selecting predictors should be clarified or strengthened with theory-driven variable selection. Figure 1 Y axis – what does 1-specifity term mean?

The manuscript should address clustering from the multistage sampling design. If clustering was not incorporated into the regression analysis, standard errors and p-values may be inaccurate.

Where is the random forest variable results described? I solicit a thorough and rigorous analyses of all the results the authors submitted for evaluation in the form of figures and tables. The results section was carelessly done..

Data availability statements and relevant consent/IRB statements are missing. Again, please refer to relevant epidemiology references to format the manuscript.

Please check all tables and figures, update legends and check consistency. In Statistical Analysis section of methods line 155 – why p<0.1 was considered?

**********

what does this mean?). If published, this will include your full peer review and any attached files.

If you choose “no”, your identity will remain anonymous but your review may still be made public.

**Do you want your identity to be public for this peer review?**  For information about this choice, including consent withdrawal, please see our Privacy Policy

Reviewer #1: **Yes:**  Alaa Jabbar Abd Al-Manhel

Reviewer #2: **Yes:**  Dr. Gosa, Asfaw Shaka

Reviewer #3: **Yes:**  Joydeep Chakraborty

**********

[NOTE: If reviewer comments were submitted as an attachment file, they will be attached to this email and accessible via the submission site. Please log into your account, locate the manuscript record, and check for the action link "View Attachments". If this link does not appear, there are no attachment files.]

To ensure your figures meet our technical requirements, please review our figure guidelines: https://journals.plos.org/plosone/s/figures

You may also use PLOS’s free figure tool, NAAS, to help you prepare publication quality figures: https://journals.plos.org/plosone/s/figures#loc-tools-for-figure-preparation.

NAAS will assess whether your figures meet our technical requirements by comparing each figure against our figure specifications.

# Author comment: Author response to Decision Letter 1

**DOI:** 10.1371/journal.pone.0361131.r002

13 Jul 2026

Manuscript ID: PONE-D-26-09587

Dear Academic Editor of PLoS ONE,

At first, we should express our special thanks to you and the reviewers for the valuable and helpful comments. Hereby, it should be mentioned that we read the comments very carefully and made substantial changes in the text.

Following the reviewers’ suggestions, the analytical pipeline was substantially revised to improve methodological rigor and reduce the risk of overfitting. Specifically, the revised analysis incorporated repeated cross-validation within the training dataset, independent test-set evaluation, transparent hyperparameter tuning, calibration assessment, Brier score evaluation, and a pre-specified prevalence-based classification threshold. After implementation of these stricter validation procedures, the relative performance of the models changed slightly compared with the initial submission. In the revised analyses, logistic regression and neural network models demonstrated marginally better overall discrimination and calibration performance than the random forest model on the independent test dataset. This finding is methodologically plausible given the characteristics of the present dataset, which primarily consisted of categorical socioeconomic predictors with relatively stable and interpretable associations with food insecurity. In such settings, simpler and more parsimonious models may generalize as well as or better than more flexible ensemble approaches. Importantly, the revised results emphasize that more complex machine learning algorithms do not necessarily outperform conventional statistical models in all epidemiologic prediction settings.

The manuscript has been revised accordingly to avoid overstating the superiority of any individual machine learning approach.

Response to the Editor's comments:

Comment 1: Please ensure that your manuscript meets PLOS ONE's style requirements, including those for file naming.

Response:

The revision has been made according to the editor's comment.

Comment 2: Please include your tables as part of your main manuscript and remove the individual files. Please note that supplementary tables (should remain/ be uploaded) as separate "supporting information" files.

Response:

All tables were included in the text according to the editor's comment.

Comment 3: We note that you have indicated that there are restrictions to data sharing for this study. For studies involving human research participant data or other sensitive data, we encourage authors to share de-identified or anonymized data. However, when data cannot be publicly shared for ethical reasons, we allow authors to make their data sets available upon request.

Response:

The data is now available upon request (line 480-483).

Comment 4: Ethics statement should only appear in Methods.

Response:

The ethics statement has been moved to the Methods section according to the editor's comment (line 257-261).

Response to reviewers #1:

Comment 1: Similar studies applying ML to food insecurity prediction already exist in public health literature.

Response:

Thank you for this valuable comment. We agree that previous studies have applied machine learning (ML) methods for food insecurity (FI) prediction and classification in different public health settings. However, the primary contribution of the current study is not merely the application of ML techniques, but rather the comparative evaluation of conventional statistical approaches and multiple ML algorithms within a large population-based dataset from northern Iran. Previous Iranian studies have mainly focused on identifying determinants of FI using conventional statistical methods, particularly logistic regression models and cross-sectional analytical approaches (1,2). In contrast, evidence regarding the simultaneous application and comparison of ML and traditional statistical approaches for identifying FI risk indicators and classifying households remains limited in the Iranian population.

To address the reviewer’s concern and avoid overstating the novelty of the study, we revised the Introduction section to better position the present work within the existing literature and clarified that the study contributes additional evidence regarding the applicability and comparative performance of different analytical approaches for FI risk assessment and classification in an Iranian population-based setting.

1. Honarvar MR, Gholami M, Abdollahi Z, Ghotbabadi FS, Lashkarboluki F, Najafzadeh M, et al. Household food insecurity and associated factors in the Northeast of Iran: a cross-sectional study: Household food security in Northern Iran. BMC nutrition. 2023;9(1):5.

2. Khodabakhshzadeh S, Moradabadi SA, Khodabakhshzadeh S, Baniasadi M, Khalilabad TH. Food security and its related factors in the rural households of Bam city in Iran: a logistic regression model approach. Archives of Iranian medicine. 2018;21(1):26-8.

Comment 2: The manuscript mainly confirms known socioeconomic determinants (income, education, employment).

Response:

We thank the reviewer for this valuable comment. The primary aim of the present study was not to establish novel socioeconomic determinants of FI, but rather to evaluate and compare the performance of conventional statistical approaches and machine learning algorithms for identifying and classifying FI risk factors within a large population-based dataset from northern Iran.

Comment 3: ML is used primarily for prediction rather than generating new theoretical insights.

Response:

We thank the reviewer for this important conceptual comment. We revised the manuscript according to the reviewer's comment.

Comment 4: Moderate originality, incremental contribution rather than innovative advancement.

Response:

We thank the reviewer for this thoughtful assessment. In this manuscript, we did not aim to present an innovative advancement in this field. Instead, our main aims were to use a large sample data for identifying some of the most important indicators of food insecurity and employ these indicators to present a framework based on both the classic and ML methodologies for predicting FI in Iranian population.

Comment 5: The manuscript does not sufficiently emphasize the limitation of causal inference.

Response:

We thank the reviewer for highlighting this important methodological point. We agree that, given the cross-sectional design of the study, causal relationships cannot be inferred from the observed associations. The findings should therefore be interpreted as associations rather than causal effects.

Comment 6: Sampling strategy is insufficiently described (randomization, clustering, weighting).

Response:

We thank the reviewer for highlighting the need for a clearer description of the sampling strategy. The Study design section has been revised according to the reviewer's comment (Lines 127–146 & 180–186).

Comment 7: Representativeness of Golestan Province relative to Iran is not justified.

Response:

We thank the reviewer for this important comment regarding the generalizability of our findings. We acknowledge that Golestan Province may not be fully representative of the entire Iranian population, given the country’s geographic, socioeconomic, and cultural diversity. However, Golestan Province is characterized by substantial heterogeneity in terms of ethnicity, socioeconomic status, and living conditions, which makes it a fairly representative sample of all Iranians and valuable setting for studying food insecurity in a diverse population.

Comment 8: No discussion of cultural validation or reliability testing within this dataset.

Response:

We thank the reviewer for this important comment regarding the validity and reliability of the measurement tool. Additional information regarding the validity and reliability of the HFIAS has been added to the Methods section (Lines 149–152).

Comment 9: Machine Learning Methodology.

This is the most critical methodological weakness.

Response:

We thank the reviewer for this important and constructive comment. We have revised the manuscript to provide a transparent and reproducible machine learning workflow (Lines 191–256).

Comment 10: Missing or unclear details:

Data preprocessing steps.

Handling of missing data.

Feature scaling or normalization.

Hyperparameter tuning strategy.

Overfitting prevention measures.

Without these details, reproducibility is limited.

Response:

We thank the reviewer for this important and constructive comment. The Statistical Analysis section has been extensively revised.

Comment 11: Accuracy alone is insufficient for imbalanced datasets.

Response:

Thank you for this comment. In addition to accuracy, we report sensitivity, specificity, positive predictive value (PPV), negative predictive value (NPV), balanced accuracy, area under the receiver operating characteristic curve (AUC) with 95% confidence intervals, and Brier score for all machine learning models. We also added calibration plots to evaluate agreement between predicted probabilities and observed event rates.

Furthermore, stratified sampling was used during both train–test splitting and repeated cross-validation procedures to preserve the original class distribution across datasets. Because the degree of class imbalance was not extreme, and to maintain the real-world distribution of food insecurity outcomes, no artificial class balancing techniques were applied.

Comment 12: No calibration metrics (Brier score, calibration plots).

Response:

We thank the reviewer for this valuable comment. We revised the manuscript according to the reviewer's comment.

Comment 13: No external validation dataset.

Response:

We thank the reviewer for this important comment. The authors are aware of the importance of assessing the external validity of the methods. However, lack of access to similar data in our country makes it impossible to evaluate the external validity of the utilized models in other datasets. We mentioned this limitation in the discussion section (line 444–450).

Comment 14: Interpretation sometimes implies explanatory power rather than predictive association.

Response:

We thank the reviewer for this important comment. We revised the text according to the reviewer's comment.

Comment 15: No comparison of effect sizes between ML and regression outputs.

Response:

We thank the reviewer for this insightful comment. Because most machine learning algorithms do not produce directly interpretable effect size estimates comparable to odds ratios, predictor importance measures derived from machine learning models were compared qualitatively with adjusted odds ratios obtained from logistic regression.

Most machine learning algorithms used in the present study are prediction-oriented rather than inference-oriented and therefore do not generate directly interpretable effect size estimates comparable to odds ratios derived from logistic regression models. To address the reviewer’s concern regarding variable influence across modeling approaches, we compared adjusted odds ratios from multivariable logistic regression with model-specific variable importance measures obtained from machine learning algorithms. This comparison was intended to evaluate the consistency of predictor relevance across conventional statistical modeling and machine learning frameworks rather than to establish direct equivalence between odds ratios and machine learning importance metrics.

Comment 16: Lack of practical policy translation.

Response:

We thank the reviewer for this valuable comment. The Discussion section have been expanded to include more explicit and actionable policy implications.

Comment 17: Confusion between prediction and inference. ML models predict outcomes but do not establish causality.

Response:

We thank the reviewer for this important clarification. The Introduction, Methods, Results, and Discussion sections have been revised to clearly distinguish predictive modeling from causal inference and to avoid any implication of causality.

Comment 18: Claim of “superior performance”. Improvement over traditional methods appears modest and not statistically tested.

Response:

We thank the reviewer for this important comment. The authors confirm that the predictive performance of the employed models are very close and the differences between estimated indices are rather insignificant. In the original manuscript, the term “superior performance” may have overstated the observed differences. We have revised the manuscript to use more cautious and accurate language, such as "slightly higher", when describing model results. We added this explanation & Delong's test in the text.

Comment 19: No uncertainty quantification for ML variable importance. Importance rankings may be unstable.

Response:

We thank the reviewer for this important methodological comment. In the revised manuscript, we expanded the analysis to address uncertainty and stability of predictor importance estimates. Specifically, for the logistic regression model, bootstrap resampling (200 iterations) was performed to estimate selection frequency, mean importance values, and bootstrap-based for predictors. This approach was incorporated to evaluate the robustness and stability of variable importance rankings across repeated samples. In addition, the revised analyses demonstrated that conventional logistic regression achieved predictive performance comparable to several machine-learning models, with only modest differences in discrimination metrics.

Response to reviewers #2:

Abstract

Comment 1: The abstract should present the key econometric analysis results more clearly and precisely to enhance its attractiveness and informativeness for readers.

Response:

We thank the reviewer for this insightful comment.

Introduction

Comment 2: In paragraph 2, the statement: “According to reports in 2023, approximately two billion people (about 28.9% of the world's population) experience moderate to severe food insecurity” requires proper citation. Please clearly indicate the source of this data to ensure its validity.

Response:

We thank the reviewer for this insightful comment. We have now added an appropriate citation to support the global food insecurity estimates reported in the Introduction.

Comment 3: A similar issue appears in paragraph 4, where claims are made without appropriate references.

Response:

We thank the reviewer for this insightful comment. We carefully reviewed paragraph 4 and sentences that required references.

Comment 4: In paragraph 5, you mention that “various studies” have identified key determinants of food insecurity, yet no specific studies are cited. At least a few representative and relevant references should be included to support this statement. Overall, the introduction should also highlight the methodological novelty of the study and clearly distinguish it from previous research.

Response:

We thank the reviewer for this insightful comment.

Methods

Comment 5: In the methodology section, it is recommended to include the sample size determination method along with the relevant formula to improve transparency and reproducibility. Additionally, a clear and concise description of the study area is essential and should be included.

Response:

We thank the reviewer for this insightful comment (Line 131).

Variables Used

Comment 6: It would be helpful to present all variables in a table, including their definitions and expected signs (hypotheses), to improve clarity and readability.

Response:

We thank the reviewer for this insightful comment.

Data Analysis

Comment 7: The section titled “Statistical Analysis” includes econometric methods such as univariate and multivariate logistic regression models. Therefore, the title should be revised to accurately reflect the econometric nature of the analysis.

Response:

We thank the reviewer for this insightful comment.

Results and Discussion

Comment 8: While the results are presented and interpreted, they should be supported by comparisons with existing literature. Engaging with other studies will strengthen the discussion and enhance the credibility of the findings. The discussion section should go beyond description and provide deeper analytical insights, clearly articulating the scientific and policy implications of the findings.

Response:

We thank the reviewer for this insightful comment.

Conclusion

Comment 9: The conclusion should clearly present the core message of the study and provide a concise synthesis of the main findings. It should not be combined with recommendations. Please present the conclusion in paragraph form and avoid bullet points.

Recommendations

The recommendations should be specific and actionable, clearly indicating what should be done, how it can be implemented, and which stakeholders are responsible for each action.

Response:

We thank the reviewer for this insightful comment.

References

Comment 10: The referencing style does not follow the journal guidelines (PLOS ONE). In-text citations should be presented using numbered brackets, whereas the current manuscript uses parenthetical citations. This should be corrected to align with the journal’s requirements.

Response:

We thank the reviewer for this insightful comment.

Response to reviewers #3:

Comment 1: Its relevance and overarching goal as contributed to the scientific community has to be clearly laid out to match the scope of PLOS one, which has a broad readership.

Response:

We thank the reviewer for this insightful comment. To match the scope of journal, we made some changes and corrections in the text.

Comment 2: Please consider a justification for the use of machine-learning methods beyond logistic regression. The predictive gain over traditional modeling appears modest, so the authors should explain why ML adds meaningful value in this dataset.

Response:

We thank the reviewer for this insightful comment. Comparing the results of traditional and machine learning methods in predicting food insecurity was one of the main objectives in the proposal of the current study. We aimed to investigate and compare the predictive performance of classic and ML methods in this data set. Since this proposal is part of Ph.D thesis in department of biostatistics, in the next phases we aim to develop innovative machine learning approaches for the analysis of similar data sets considering both the main and interaction effects of predictors in the modeling process.

Comment 3: Methods section requires significant improvement. The analysis workflow for the machine-learning models is insufficiently described. The authors should report train/test partitioning, cross-validation strategy, hyperparameter tuning, handling of class imbalance, and whether performance metrics were evaluated on independent data. Overall for clarity the programs used for this work and their licenses have to be clearly stated. Please refer to relevant literature.

Response:

We thank the reviewer for this insightful comment. We substantially revised the Methods section according to the reviewer's comment.

Comment 4: I am skeptical about the authors over interpreting their cross-sectional study. Abbreviation jargons must be decoded in the tables and figures’ legends. For example Table 3. including HFIAS, RF, NN, SVM, NB, DT, PPV, NPV, AUC, and ACC.

Response:

We thank the reviewer for this insightful comment. We carefully revised the manuscript to avoid over interpretation of findings from this cross-sectional study and to maintain a predictive rather than causal interpretation throughout the manuscript. Specifically, statements implying superiority or causal inference were moderated and replaced with more cautious language emphasizing comparative predictive performance. In addition, all abbreviations used in tables and figure legends have now been fully defined for clarity and readability.

Comment 5: The model-selection strategy needs more rigor. The use of univariate screening followed by multivariable modeling may introduce bias, and the rationale for selecting predictors should be clarified or strengthened with theory-driven variable selection.

Response:

We thank the reviewer for this insightful comment. The multivariable analysis was restructured using forward stepwise logistic regression based on the Akaike Information Criterion (AIC), rather than relying solely on univariate statistical significance for selecting the predictors.

Comment 6: Figure 1 Y axis – what does 1-specifity term mean?

Response:

We thank the reviewer for this insightful comment. In receiver operating characteristic (ROC) analysis, the term “1-specificity” refers to the false positive rate, which represents the proportion of non-events incorrectly classified as events by the prediction model. In the context of the present study, it indicates the proportion of food-secure households incorrectly classified as food insecure. The use of sensitivity on the y-axis and 1-specificity on the x-axis is the conventional and standard presentation of ROC curves in diagnostic and predictive modeling studies. We retained this terminology to maintain consistency with established ROC methodology.

Comment 7: The manuscript should address clustering from the multistage sampling design. If clustering was not incorporated into the regression analysis, standard errors and p-values may be inaccurate.

Response:

We thank the reviewer for this insightful comment. We reanalyzed the data to incorporate the weighting factors in the model fitting process. The process was explained in the Methods section.

Comment 8: Where is the random forest variable results described? I solicit a thorough and rigorous analyses of all the results the authors submitted for evaluation in the form of figures and tables. The results section was carelessly done.

Response:

We thank the reviewer for this insightful comment. In table 5, we added some descriptions about the output of random forest analysis including variable importance rankings and reporting the correlation between these ranks with those obtained from the multivariable logistic regression model.

Comment 9: Data availability statements and relevant consent/IRB statements are missing. Again, please refer to relevant epidemiology references to format the manuscript.

Response:

We thank the reviewer for this insightful comment. We added the data availability statement in the text.

Comment 10: Please check all tables and figures, update legends and check consistency.

Response:

We thank the reviewer for this insightful comment. We rechecked the tables and figures.

Comment 11: In Statistical Analysis section of methods line 155 – why p<0.1 was considered?

Response:

We thank the reviewer for this insightful comment. We included the results from fitting the multivariable logistic regression with forward selection method instead of screening the variables using the univariate analysis.

Attachment Submitted filename: Response to Reviewers.docx

# Peer review: Decision Letter 1

**DOI:** 10.1371/journal.pone.0361131.r003

**Authors:** Jamil Afzal

26 Aug 2026

Dear Dr. Zayeri,

Thank you for submitting your manuscript to PLOS One. After careful consideration, we feel that it has merit but does not fully meet PLOS One’s publication criteria as it currently stands. Therefore, we invite you to submit a revised version of the manuscript that addresses the points raised during the review process.

Please submit your revised manuscript by Oct 10 2026 11:59PM. If you will need more time than this to complete your revisions, please reply to this message or contact the journal office at plosone@plos.org. When you're ready to submit your revision, log on to https://www.editorialmanager.com/pone/ and select the 'Submissions Needing Revision' folder to locate your manuscript file.

- A letter that responds to each point raised by the academic editor and reviewer(s). You should upload this letter as a separate file labeled 'Response to Reviewers'.
- A marked-up copy of your manuscript that highlights changes made to the original version. You should upload this as a separate file labeled 'Revised Manuscript with Track Changes'.
- An unmarked version of your revised paper without tracked changes. You should upload this as a separate file labeled 'Manuscript'.

If you would like to make changes to your financial disclosure, please include your updated statement in your cover letter. Guidelines for resubmitting your figure files are available below the reviewer comments at the end of this letter.

If applicable, we recommend that you deposit your laboratory protocols in protocols.io to enhance the reproducibility of your results. Protocols.io assigns your protocol its own identifier (DOI) so that it can be cited independently in the future. For instructions see: https://journals.plos.org/plosone/s/submission-guidelines#loc-laboratory-protocols. Additionally, PLOS One offers an option for publishing peer-reviewed Lab Protocol articles, which describe protocols hosted on protocols.io. Read more information on sharing protocols at https://plos.org/protocols?utm_medium=editorial-email&utm_source=authorletters&utm_campaign=protocols.

As the corresponding author, your ORCID iD is verified in the submission system and will appear in the published article. PLOS supports the use of ORCID, and we encourage all coauthors to register for an ORCID iD and use it as well. Please encourage your coauthors to verify their ORCID iD within the submission system before final acceptance, as unverified ORCID iDs will not appear in the published article. *Only*  the individual author can complete the verification step; PLOS staff *cannot*  verify ORCID iDs on behalf of authors.

We look forward to receiving your revised manuscript.

Kind regards,

Jamil Afzal, Ph.D, Post Doc

Academic Editor

PLOS One

Journal Requirements:

1. If the reviewer comments include a recommendation to cite specific previously published works, please review and evaluate these publications to determine whether they are relevant and should be cited. There is no requirement to cite these works unless the editor has indicated otherwise.

2. Please review your reference list to ensure that it is complete and correct. If you have cited papers that have been retracted, please include the rationale for doing so in the manuscript text, or remove these references and replace them with relevant current references. Any changes to the reference list should be mentioned in the rebuttal letter that accompanies your revised manuscript. If you need to cite a retracted article, indicate the article’s retracted status in the References list and also include a citation and full reference for the retraction notice.

Additional Editor Comments:

Overall, the major reviewer concerns have been substantially addressed. However, I would not recommend acceptance without further revision.

• The manuscript states that missing observations were handled through complete-case analysis because the missing rate was below 6%. However, the mechanism of missingness (MCAR, MAR, or potentially MNAR) is not sufficiently demonstrated.

• The manuscript uses a fixed threshold of 0.61 for all classification models because it approximately reflects the observed prevalence of food insecurity. However, prevalence matching does not necessarily establish that 0.61 is the optimal or clinically/policy-relevant threshold.

• The authors should consider reporting a quantitative calibration measure, such as calibration intercept and calibration slope, and explain which model demonstrates the best calibration. This is particularly important because the study emphasizes prediction rather than merely discrimination.

• The authors should distinguish between internal validation through the 30% test set and genuine external validation in another geographic population/time period.

• The conclusion should focus on the principal empirical findings, while policy recommendations should be presented separately and expressed cautiously.

[Note: HTML markup is below. Please do not edit.]

Reviewer's Responses to Questions

**Comments to the Author**

Reviewer #3: All comments have been addressed

**********

2. Is the manuscript technically sound, and do the data support the conclusions?

Reviewer #3: Yes

**********

3. Has the statistical analysis been performed appropriately and rigorously?

Reviewer #3: Yes

**********

4. Have the authors made all data underlying the findings in their manuscript fully available?

The PLOS Data policy

Reviewer #3: Yes

**********

5. Is the manuscript presented in an intelligible fashion and written in standard English?

Reviewer #3: Yes

**********

Reviewer #3: Thank you for the thorough revision of your manuscript and addressing all concerns carefully. I recommend accept for publication.

**********

what does this mean?). If published, this will include your full peer review and any attached files.

If you choose “no”, your identity will remain anonymous but your review may still be made public.

**Do you want your identity to be public for this peer review?**  For information about this choice, including consent withdrawal, please see our Privacy Policy

Reviewer #3: **Yes:**  Joydeep Chakraborty

**********

[NOTE: If reviewer comments were submitted as an attachment file, they will be attached to this email and accessible via the submission site. Please log into your account, locate the manuscript record, and check for the action link "View Attachments". If this link does not appear, there are no attachment files.]

To ensure your figures meet our technical requirements, please review our figure guidelines: https://journals.plos.org/plosone/s/figures

You may also use PLOS’s free figure tool, NAAS, to help you prepare publication quality figures: https://journals.plos.org/plosone/s/figures#loc-tools-for-figure-preparation.

NAAS will assess whether your figures meet our technical requirements by comparing each figure against our figure specifications.

# Author comment: Author response to Decision Letter 2

**DOI:** 10.1371/journal.pone.0361131.r004

28 Sep 2026

Manuscript ID: PONE-D-26-09587R1

Dear Academic Editor of PLoS ONE,

We sincerely appreciate your careful review of our manuscript and your constructive and insightful comments. We are grateful for the opportunity to further improve the manuscript in response to your recommendations. We have carefully considered each comment and have revised the manuscript accordingly. A detailed, point-by-point response to the Editor's comments is provided below.

Response to the Editor's comments:

Comment 1: The manuscript states that missing observations were handled through complete-case analysis because the missing rate was below 6%. However, the mechanism of missingness (MCAR, MAR, or potentially MNAR) is not sufficiently demonstrated.

Response:

We thank the Academic Editor for this important observation. In the revised manuscript, we have expanded the description of missing-data handling. Prior to conducting complete-case analysis, we examined the pattern and extent of missingness across variables. The overall proportion of missing data was low (below 6% for all variables), and no clear evidence of systematic differences in missingness across the primary outcome categories or key demographic characteristics was observed. Given the limited amount of missing information and the absence of evidence suggesting substantial informative missingness, complete-case analysis was retained as the primary approach. We have clarified this rationale in the Statistical Analysis section.

Comment 2: The manuscript uses a fixed threshold of 0.61 for all classification models because it approximately reflects the observed prevalence of food insecurity. However, prevalence matching does not necessarily establish that 0.61 is the optimal or clinically/policy-relevant threshold.

Response:

We appreciate this valuable comment. We agree that a prevalence-based threshold (fixed threshold of 0.61) does not necessarily represent the optimal decision threshold for clinical or policy applications. The primary objective of this study was to compare the predictive performance of different modeling approaches under a common evaluation framework rather than to develop a decision-support tool with a specific operational threshold. Therefore, a fixed threshold corresponding approximately to the observed prevalence of food insecurity was applied uniformly across all models to facilitate direct comparison of classification metrics. Importantly, the primary performance measure used for model comparison was based on the Youden index in ROC curve analysis, which is independent of any particular classification threshold.

Comment 3: The authors should consider reporting a quantitative calibration measure, such as calibration intercept and calibration slope, and explain which model demonstrates the best calibration. This is particularly important because the study emphasizes prediction rather than merely discrimination.

Response:

Thank you for this valuable suggestion. In the revised manuscript, we supplemented the calibration plots with quantitative calibration measures, including calibration intercepts and calibration slopes, calculated using the independent test dataset. The results have been added as Supplementary Table 2.

Comment 4: The authors should distinguish between internal validation through the 30% test set and genuine external validation in another geographic population/time period.

Response:

We thank the Academic Editor for highlighting this important issue. In the revised manuscript, we have clarified that the independent 30% test dataset represents an internal validation procedure because it was derived from the same source population as the training dataset. We explicitly distinguish this approach from external validation, which requires evaluation in an independent population, geographic region, or time period. We have revised the Methods, Results, and Limitations sections accordingly and emphasize that external validation was not performed in the present study.

Comment 5: The conclusion should focus on the principal empirical findings, while policy recommendations should be presented separately and expressed cautiously.

Response:

We appreciate this recommendation. In the revised manuscript, the Conclusion section has been rewritten to focus primarily on the main empirical findings of the study, including the prevalence of food insecurity, the identified socioeconomic predictors, and the comparative performance of the evaluated predictive models. Policy implications have been moved to the Discussion section and rephrased more cautiously to reflect their exploratory and contextual nature.

Attachment Submitted filename: Response_to_Reviewers_auresp_2.docx

# Editor report: Decision Letter 2

**DOI:** 10.1371/journal.pone.0361131.r005

**Authors:** Jamil Afzal

7 Oct 2026

Food Insecurity and its Related Indicators in an Iranian Population: an application of machine learning methods in data from Golestan Province

PONE-D-26-09587R2

Dear Dr. Zayeri,

We’re pleased to inform you that your manuscript has been judged scientifically suitable for publication and will be formally accepted for publication once it meets all outstanding technical requirements.

Within one week, you’ll receive an e-mail detailing the required amendments. When these have been addressed, you’ll receive a formal acceptance letter and your manuscript will be scheduled for publication.

An invoice will be generated when your article is formally accepted. Please note, if your institution has a publishing partnership with PLOS and your article meets the relevant criteria, all or part of your publication costs will be covered. Please make sure your user information is up-to-date by logging into Editorial Manager at Editorial Manager® and clicking the ‘Update My Information' link at the top of the page. For questions related to billing, please contact billing support.

If your institution or institutions have a press office, please notify them about your upcoming paper to help maximize its impact. If they’ll be preparing press materials, please inform our press team as soon as possible -- no later than 48 hours after receiving the formal acceptance. Your manuscript will remain under strict press embargo until 2 pm Eastern Time on the date of publication. For more information, please contact onepress@plos.org.

Kind regards,

Jamil Afzal, Ph.D, Post Doc

Academic Editor

PLOS One

Additional Editor Comments (optional):

Reviewers' comments:

# Editor report: Acceptance letter

**DOI:** 10.1371/journal.pone.0361131.r006

**Authors:** Jamil Afzal

PONE-D-26-09587R2

PLOS One

Dear Dr. Zayeri,

I'm pleased to inform you that your manuscript has been deemed suitable for publication in PLOS One. Congratulations! Your manuscript is now being handed over to our production team.

At this stage, our production department will prepare your paper for publication. This includes ensuring the following:

* All references, tables, and figures are properly cited

* All relevant supporting information is included in the manuscript submission,

* There are no issues that prevent the paper from being properly typeset

You will receive further instructions from the production team, including instructions on how to review your proof when it is ready. Please keep in mind that we are working through a large volume of accepted articles, so please give us a few days to review your paper and let you know the next and final steps.

Lastly, if your institution or institutions have a press office, please let them know about your upcoming paper now to help maximize its impact. If they'll be preparing press materials, please inform our press team within the next 48 hours. Your manuscript will remain under strict press embargo until 2 pm Eastern Time on the date of publication. For more information, please contact onepress@plos.org.

You will receive an invoice from PLOS for your publication fee after your manuscript has reached the completed accept phase. If you receive an email requesting payment before acceptance or for any other service, this may be a phishing scheme. Learn how to identify phishing emails and protect your accounts at https://explore.plos.org/phishing.

If we can help with anything else, please email us at customercare@plos.org.

Thank you for submitting your work to PLOS One and supporting open access.

Kind regards,

PLOS One Editorial Office Staff

on behalf of

Dr. Jamil Afzal

Academic Editor

PLOS One
